# Genome-wide conditional association study reveals the influences of lifestyle cofactors on genetic regulation of body surface area in MESA population

**DOI:** 10.1371/journal.pone.0253167

**Published:** 2021-06-18

**Authors:** Mita Khatun, Md. Mamun Monir, Ting Xu, Haiming Xu, Jun Zhu

**Affiliations:** 1 Institute of Bioinformatics, Zhejiang University, Hangzhou, China; 2 Department of Mathematics, Zhejiang University, Hangzhou, China; Illumina Inc, UNITED STATES

## Abstract

Body surface area (BSA) is an important trait used for many clinical purposes. People’s BSA may vary due to genetic background, race, and different lifestyle factors (such as walking, exercise, reading, smoking, transportation, etc.). GWAS of BSA was conducted on 5,324 subjects of four ethnic populations of European-American, African-American, Hispanic-American, and Chinese-American from the Multi-Ethnic Study of Atherocloris (MESA) data using unconditional and conditional full genetic models. In this study, fifteen SNPs were identified (Experiment-wise *P*_*EW*_ < 1×10^−5^) using unconditional full genetic model, of which thirteen SNPs had individual genetic effects and seven SNPs were involved in four pairs of epistasis interactions. Seven single SNPs and eight pairs of epistasis SNPs were additionally identified using exercise, smoking, and transportation cofactor-conditional models. By comparing association analysis results from unconditional and cofactor conditional models, we observed three different scenarios: (i) genetic effects of several SNPs did not affected by cofactors, e.g., additive effect of gene *CREB5* (*a*≙ –0.013 for T/T and 0.013 for G/G, −*Log*_10_ *P*_*EW*_ = 8.240) did not change in the cofactor models; (ii) genetic effects of several SNPs affected by cofactors, e.g., the genetic additive effect (*a*≙ 0.012 for A/A and –0.012 for G/G, −*Log*_10_ *P*_*EW*_ = 7.185) of SNP of the gene *GRIN2A* was not significant in transportation cofactor model; and (iii) genetic effects of several SNPs suppressed by cofactors, e.g., additive (*a*≙ –0.018 for G/G and 0.018 for C/C, −*Log*_10_ *P*_*EW*_ = 19.737) and dominance (*d*≙ –0.038 for G/C, −*Log*_10_ *P*_*EW*_ = 27.734) effects of SNP of gene *ERBB4* was identified using only transportation cofactor model. Gene ontology analysis showed that several genes are related to the metabolic pathway of calcium compounds, coronary artery disease, type-2 Diabetes, Alzheimer disease, childhood obesity, sleeping duration, Parkinson disease, and cancer. This study revealed that lifestyle cofactors could contribute, suppress, increase or decrease the genetic effects of BSA associated genes.

## Introduction

Body surface area (BSA) is the measured surface area of human body used for many clinical purposes in physiology and medicine [1]. It is associated with several diseases including cardiovascular diseases and cancer. There was an average BSA of 1.73 *m*^2^ for 3,000 cancer patients from 1990 to 1998 in a European Organization for Research and Treatment of Cancer (EORTC) database [2], and during 2005 there was an average BSA of 1.79 *m*^2^ for 3,613 adult cancer patients in the UK [3]. Among them, the average BSA was 1.91 *m*^2^ for men and 1.71 *m*^2^ for women. Weight gain can be a specific surrogate marker of obesity, which can contribute to cancer development [4]. Long-term weight gain or increasing BSA is considered as a factor for assessing the risk of papillary thyroid cancer [4]. Values of BSA are commonly used in medicine as the biometric unit for normalizing physiologic parameters, such as cardiac output, left ventricular mass, renal clearance; and for the determination of appropriate drug dosages in cancer chemotherapy [1,5]. It can determine the efficacy of a drug. A recent study with prospective cohort data showed that BSA could affect the efficacy of gefitinib monotherapy (an approved dose) in patients with EGFR-mutant non-small cell lung cancer [6]. It is a predictor of coronary artery calcium [7], and related disease coronary artery disease (CAD) is the foremost cause of death in many countries [8].

In the area of quantitative genetics, it is well known that complex traits are controlled by multiple genes, epistasis, and gene-environment interactions [9–11]. Therefore, multiple genetic variants and environmental modulators may control BSA. Unconditonal and conditional full genetic models with genetic effects of additive, dominance, epistasis, and gene-environment interaction were used for analyzing BSA. In this study, different self-reported ethnicities of individuals of the MESA population were used as environments for analyzing gene-environment interactions. Lifestyle cofactors could have influence on this complex trait. Some of the cofactors may expend consuming calories of the human body and help in reducing the risk of obesity. A number of publications suggested that lifestyles have influences on obesity [12–16]. In this study, GWAS of BSA was analyzed using the unconditional and conditional full genetic models with additive, dominance, epistasis, and their ethnic-specific effects for investigating the impacts of lifestyle factors on genetic effects of genes that regulate BSA Five different lifestyle cofactors, such as walking, exercise, reading, smoking, and transportation were used in conditional GWAS models for dissecting the complex genetic architecture of BSA and investigating the impacts of lifestyle cofactors on detected SNPs. This study revealed an overview of the genetic mechanism of BSA and impact of lifestyle cofactors in the MESA population.

## Materials and methods

### Data

Genotypes, phenotypes, and cofactors data sets were obtained from the Multi-Ethnic Study of Atherosclerosis (MESA) downloaded from dbGaP (database of Genotypes and Phenotypes, http://www.ncbi.nlm.nih.gov/gap) [17]. MESA is a prospective population-based study focusing on characterization of subclinical cardiovascular disease and the risk factors that enable prediction of the progression of CVD. Study participants of four ethnic groups include 6,500 men and women, nearly in equal numbers, who are aged 45~84 years and free of clinical CVD at baseline, and initially recruited in 2000 from six US communities: Baltimore, MD; Chicago, IL, Forsyth County, NC; Los Angeles County, CA, Northern Manhattan, NY; and St. Paul, MN. The recruited participants are approximately 38% European-American (E-A), 28% African-American (A-A), 22% Hispanic-American (H-A), and 12% Chinese-American (C-A). We observed that averages of BSA in the four ethnic groups were different: smallest for C-A (mean = 1.667 m^2^; 95% CI = (1.654, 1.681)) and largest for A-A (mean = 1.958 m^2^; 95% CI = (1.654, 1.681)). Averages of BSA were 1.901 m^2^ (95% CI = (1.892, 1.911)) for E-A and 1.812 m^2^ (95% CI = (1.801, 1.823)) for H-A. BSA of different ethnic groups had diverse distributions, which varied based on mean and variance (S1D Fig).

Phenotype and five different lifestyle cofactors were used for conditional GWAS analysis: Moderate Walking (walking to get places to the bus, car, work, into the store; minute/week), Moderate Walking Exercise (min/wk M ~ Su), Light Leisure Read (Read, knit, sew, visit, do nothing, non-work recreational computer; minute/week), Pack-Years of Cigarette Smoking, and Light Transportation (drive or ride in car, ride the bus/subway, including travel to work; minute/week) data were used in this study. Again, significant sex differences observed within each ethnic group. Therefore, sex was used as block and ethnic effects were used as random factors to control confounding due to sex and ethnic effects in our analyses.

### Quality control

Before the analysis, we checked the quality of the genotypes, and phenotypes data. We discarded SNPs with MAF < 0.05, and call rate < 90%. Frequencies of the identified SNPs using association analyses were tabulated in S1 Table. Total, 866,435 SNPs from 22 autosomes were used for association analyses. Initially, we removed phenotypic outliers based on quartile and interqurtile range. The phentypic data larger than Q2+1.5×IQR and smaller than Q1-1.5×IQR were deleted. Moreover, phenotypic data were filtered based on standardized residual analysis. In this case, full model was fitted using the phenotypic data available after filtering based on quartile and interquartile range, and calculated residuals. Then, the phenotype was filtered based on the distribution-based abnormality detection of residues (|*ε*− *μ*_*ε*_|/*σ*_*ε*_>3). Finally, 5324 subjects were used for unconditional and conditional association analyses.

### Statistical model for association analysis

Analyses were performed by using a mixed linear model approach implemented in *QTXNetwork*. The mixed linear model includes SNP loci effects (*a*, *d*, *aa*, *ad*, *da*, *dd*) as fixed; ethnicity (*e*) and loci by ethnicity interaction (*ae*, *de*, *aae*, *ade*, *dae*, *dde*) as random effects,

$$\begin{array}{l} y_{hk}=\mu +s_{k}+c_{hk}+\sum_{i}a_{i}x_{A_{ik}}+\sum_{i}d_{i}x_{D_{ik}}+\sum_{i<j}aa_{ij}x_{AA_{ijk}}+\sum_{i<j}ad_{ij}x_{AD_{ijk}}+\sum_{i<j}da_{ij}x_{DA_{ijk}}+\sum_{i<j}dd_{ij}x_{DD_{ijk}}\\ \;+e_{h}+\sum_{i}ae_{ih}u_{AE_{ihk}}+\sum_{i}de_{ih}u_{DE_{ihk}}+\sum_{i}aae_{ih}u_{AAE_{ihk}}+\sum_{i}ade_{ih}u_{ADE_{ihk}}+\sum_{i}dae_{ih}u_{DAE_{ihk}}+\sum_{i}dde_{ih}u_{DDE_{ihk}}+\varepsilon_{hk} \end{array}$$

Where, *y*_*hk*_ is of *k*^*th*^ individual in the *h*^*th*^ ethnic group; *μ* is the population mean; *s*_*hk*_ is the sex of *k*^*th*^ individual in the *h*^*th*^ ethnic group; *c*_*hk*_ is the lifestyle cofactor of *k*^*th*^ individual in the *h*^*th*^ ethnic group; *a*_*i*_ is the additive effect of the *i*^*th*^ locus with coefficient $x_{A_{ik}}$ (1 for *QQ*, 0 for *Qq*, –1 for *qq*); *d*_*i*_ is the dominance effect of the *i*^*th*^ locus with coefficient $x_{D_{ik}}$ (1 for *Qq*, 0 for *QQ* and *qq*); *aa*_*ij*_, *ad*_*ij*_, *da*_*ij*_ and *dd*_*ij*_ are the digenic epistasis effects with coefficients $x_{AA_{ijk}}$ (1 for *QQ × QQ* and *qq × qq*, –1 for *QQ × qq* and *qq × QQ*, and 0 for others), $x_{AD_{ijk}}$ (1 for *QQ × Qq*, –1 for *qq × Qq*, and 0 for others), $x_{DA_{ijk}}$ (1 for *Qq × QQ*, –1 for *Qq × qq*, and 0 for others) and $x_{DD_{ijk}}$ (1 for *Qq ×Qq*, and 0 for others); *e*_*h*_ is the effect of the *h*^*th*^ ethnic group (1 for E-A, 2 for C-A, 3 for A-A, and 4 for H-A); *ae*_*ih*_ is the additive × ethnic interaction effect of the *i*^*th*^ locus in the *h*^*th*^ ethnic group with coefficient $u_{AE_{ihk}}$; *de*_*ih*_ is the dominance × ethnic interaction effect of the *i*^*th*^ locus in the *h*^*th*^ ethnic group with coefficient $u_{DE_{ihk}}$; *aae*_*ijh*_, *ade*_*ijh*_, *dae*_*ijh*_ and *dde*_*ijh*_ are the digenic epistasis × ethnic interaction effects in the *h*^*th*^ ethnic group with coefficient $u_{AAE_{ijhk}}$, $u_{ADE_{ijhk}}$, $u_{DAE_{ijhk}}$ and $u_{DDE_{ijhk}}$; and *ε*_*hk*_ is the residual effect of the *k*^*th*^ individual in the *h*^*th*^ ethnic group. The exact forms of unconditional and conditional genetic model written in S1 Text.

Similar to a recent study [18] heritability of individual genetic effects were estimated by $h_{g}^{2}=\alpha \sigma_{g}^{2}/V_{P}$ (*α* = 2 for aditive effect, *α* = 1 for dominant effect, *α* = 4 for additive × additive, *α* = 2 for additive × dominant or dominant × additive, *α* = 1 for dominant × dominant), where phenotypic variance (*V*_*P*_) is the sum of genetic variance (*V*_*G*_), genetic by environment interaction variance (*V*_*GE*_), and residual variance (*V*_*ε*_),

$$\begin{array}{l} V_{P}=V_{G}+V_{GE}+V_{\varepsilon }\\ \;=(V_{A}+V_{D}+V_{I})+(V_{AE}+V_{DE}+V_{IE})+V_{\varepsilon }\\ \;=(V_{A}+V_{D}+V_{AA}+V_{AD}+V_{DA}+V_{DD})+\\ \;(V_{AE}+V_{DE}+V_{AAE}+V_{ADE}+V_{DAE}+V_{DDE})+V_{\varepsilon } \end{array}$$

The total heritability is estimated by

$$\begin{array}{l} h_{T}^{2}=h_{G}^{2}+h_{GE}^{2}\\ \;=(h_{A}^{2}+h_{D}^{2}+h_{AA}^{2}+h_{AD}^{2}+h_{DA}^{2}+h_{DD}^{2})+\\ \;(h_{AE}^{2}+h_{DE}^{2}+h_{AAE}^{2}+h_{ADE}^{2}+h_{DAE}^{2}+h_{DDE}^{2})\\ \;=(h_{A}^{2}+h_{D}^{2}+h_{I}^{2})+(h_{AE}^{2}+h_{DE}^{2}+h_{IE}^{2}) \end{array}$$

where $h_{T}^{2}$ is the total heritability; $h_{A}^{2}=\sum_{i}h_{a}^{2}$ is the heritability due to additive effects contributed by sum of individual locus, $h_{D}^{2}=\sum_{i}h_{d}^{2}$ is the heritability due to dominance effects contributed by sum of individual locus, $h_{AA}^{2}=\sum_{i}h_{aa}^{2}$ is the heritability contributed by sum of pair-wise additive by additive (*aa*) epistasis, $h_{AD}^{2}=\sum_{i}h_{ad}^{2}$ is the heritability contributed by sum of pair-wise additive by dominance (*ad*) epistasis, $h_{DA}^{2}=\sum_{i}h_{da}^{2}$ is the heritability contributed by sum of pair-wise dominance by additive (*da*) epistasis, $h_{DD}^{2}=\sum_{i}h_{dd}^{2}$ is the heritability contributed by sum of pair-wise dominance by dominance (*dd*) epistasis, $h_{AE}^{2}=\sum_{i}h_{ae}^{2}$ is additive by environment interaction heritability contributed by sum of individual additive by environment interaction effects, $h_{DE}^{2}=\sum_{i}h_{de}^{2}$ is dominance by environment interaction heritability contributed by sum of individual dominance by environment interaction effects, $h_{AAE}^{2}=\sum_{i}h_{aae}^{2}$ is *aa* epistasis by environment interaction heritability contributed by sum of pair-wise *aa* epistasis by environment interaction effects, $h_{ADE}^{2}=\sum_{i}h_{ade}^{2}$ is *ad* epistasis by environment interaction heritability contributed by sum of pair-wise *ad* epistasis by environment interaction effects, $h_{DAE}^{2}=\sum_{i}h_{dae}^{2}$ is *da* epistasis by environment interaction heritability contributed by sum of pair-wise *da* epistasis by environment interaction effects, $h_{DDE}^{2}=\sum_{i}h_{dde}^{2}$ is *dd* epistasis by environment interaction heritability contributed by sum of pair-wise *dd* epistasis by environment interaction effects.

The linear mixed model with Henderson method III [19] was used to construct the F-statistic test for association analysis. Permutation test was conducted by a total of 2,000 times for calculating the critical F-value to control the experiment-wise type I error (< 0.05). The QTS effects were estimated by using the MCMC (Markov Chain Monte Carlo) algorithm with 20,000 Gibbs sample iterations [20–23]. The critical experiment-wise P value (*P*_*EW*_-value) for genetic effects by controlling the experiment-wise type I error (*P*_EW_ < 0.05) was thus calculated.

### Genetic effects analysis

R code was written for plotting the genetic effects matrix. Individuals were in the same order for both base model identified loci and other loci that only identified in the diferent lifestyle cofactor models. The principal component analysis was used for sorting individuals in each ethnic group for better presenting the pattern of genetic effects in the ethnic groups.

### Bioinformatics analysis of candidate genes corresponding to SNPs

Genes corresponding to identified SNPs from association analyses were retrived using HaploReg (https://pubs.broadinstitute.org/mammals/haploreg/haploreg.php). Bioinformatics analysis was conducted by using search engine *BiopubInfo* (http://ibi.zju.edu.cn/biopubInfo/)). We also used GeneCards (http://www.genecards.org), UniProtKB (http://www.uniprot.org/), PheGenI (https://www.ncbi.nlm.nih.gov/gap/phegeni), and Ensembl database (http://asia.ensembl.org/Homo_sapiens/) to search gene ontology, functions, and associated diseases or traits of the candidate genes.

## Results

### Estimated heritability

In GWAS, small genetic heritability for complex traits was estimated in most of the previous studies [24], referred to the problem as missing heritability. However, a recent study for LDL cholesterol of the MESA population estimated 72.88% heritability using full genetic model [25]. Like the LDL study, large heritability was also estimated for BSA using full genetic model. Base and cofactor models estimated roughly similar total heritability (74.85 ~ 79.87%), as detailed in Table 1 and S2 Fig.

**Table 1:** Estimated heritability of significant quantitative trait single nucleotide polymorphisms (QTSs) for body surface area (BSA) and five cofactor models of lifestyles.

Model	$h_{A}^{2}$	$h_{D}^{2}$	$h_{AA}^{2}$	$h_{AD}^{2}$	$h_{DA}^{2}$	$h_{DD}^{2}$	$h_{AE}^{2}$	$h_{DE}^{2}$	$h_{AAE}^{2}$	$h_{ADE}^{2}$	$h_{DAE}^{2}$	$h_{DDE}^{2}$	$h_{T}^{2}$	$h_{D_{+}}^{2}$	$h_{GE}^{2}$
**BSA**	7.06	4.24	2.27	8.33	2.43	5.21	4.23	3.38	2.37	18.76	5.83	10.74	74.85	58.92	45.31
**BSA|Walk**	7.23	4.61	2.43	8.82	2.85	5.67	4.09	3.37	2.27	18.10	5.62	10.52	75.58	59.56	43.97
**BSA|Exer**	5.12	5.97	4.02	3.36	5.74	12.71	1.73	9.95	1.78	9.34	3.43	14.58	77.73	65.08	40.81
**BSA|Read**	7.24	4.57	2.43	8.78	2.84	5.60	4.10	3.39	2.26	18.18	5.63	10.52	75.54	59.51	44.08
**BSA|Smoke**	5.54	7.14	6.68	6.82	8.02	9.09	4.27	7.74	9.40	0.54	7.22	4.43	76.89	51.00	33.6
**BSA|Trans**	6.28	4.34	5.40	3.53	6.61	3.42	3.08	11.79	7.53	9.92	5.52	12.45	79.87	57.58	50.29

BSA = Base model for body surface area with additive, dominance and epistasis effects; BSA|Walk = Conditional model for body surface area with walk as a cofactor; BSA|Exer = Conditional model for body surface area with exercise as a cofactor; BSA|Read = Conditional model for body surface area with read as a cofactor; BSA|Smoke = Conditional model for body surface area with smoke as a cofactor; BSA|Trans = Conditional model for body surface area with transportation as a cofactor; Heritability: $h_{A}^{2}$ = heritability for additive effects, $h_{D}^{2}$ = heritability for dominance effects, $h_{AA}^{2}$ = heritability for additive by additive (AA) epistasis effects; $h_{AD}^{2}$ = heritability for additive by dominance (AD) epistasis effects; $h_{DA}^{2}$ = dominance by additive (DA) epistasis effects; $h_{DD}^{2}$ = heritability for dominance by dominance (DD) epistasis effects; $h_{AE}^{2}$ = heritability for ethnicity-specific additive effects, $h_{DE}^{2}$ = heritability for ethnicity-specific dominance effects; $h_{AAE}^{2}$ = heritability for ethnicity-specific additive by additive (AA) epistasis effects; $h_{ADE}^{2}$ = heritability for ethnicity-specific additive by dominance (AD) and dominance by additive (DA) epistasis effects; $h_{DDE}^{2}$ = heritability for ethnicity-specific dominance by dominance (DD) epistasis effects; $h_{T}^{2}$ = total heritability; $h_{D+}^{2}$ = sum of the heritability’s of dominance and dominance related epistasis effects.

In the base model, it was observed that dominance, dominance-related epistasis, and dominance-related ethnic-specific genetic effects highly contributed to phenotypic variations (58.92%), which was larger as compared to additive, additive related epistasis, and additive related racial-specific genetic effects (15.93%). Association analysis revealed significant impacts of different lifestyle cofactors on heritability estimation, thus genetic variation due to identified SNPs could be largely influenced by lifestyles. Although estimates of total heritability from cofactor models were like that of the base model, their genetic composition was quite different. For example, using the transportation cofactor model, the estimated heritability caused by ethnic-specific additive × additive epistasis effects increased significantly, but heritability due to ethnic-specific additive × dominant effects reduced as compared to the base model. Moreover, total heritability due to dominance, dominance related epistasis, and dominance-related ethnicity-specific effects were similar in walk and read cofactor models, but higher in transportation cofactor model (Table 1). For example, exercise and smoking had different impacts on genetic effects. Heritability due to dominance and dominance-related epistasis effects in the exercise cofactor model was higher (65.08%) but in the smoking cofactor model was lower (51.00%). When exercise was used as a cofactor, heritability of additive and ethnic-specific additive effects decreased (6.85%). Remarkably, epistasis and race-specific genetic effects of SNPs could be largely varied by lifestyles. From S2 Fig, it can be observed that heritability due to race-specific genetic effects was larger in transportation cofactor model and smaller in smoking cofactor model. The reverse case occurs for epistasis effects in these models. Total heritability was high when the effects of transportation were removed (79.87%).

### Genetic effects of several SNPs not affected by lifestyle cofactors

**Fig 1:**
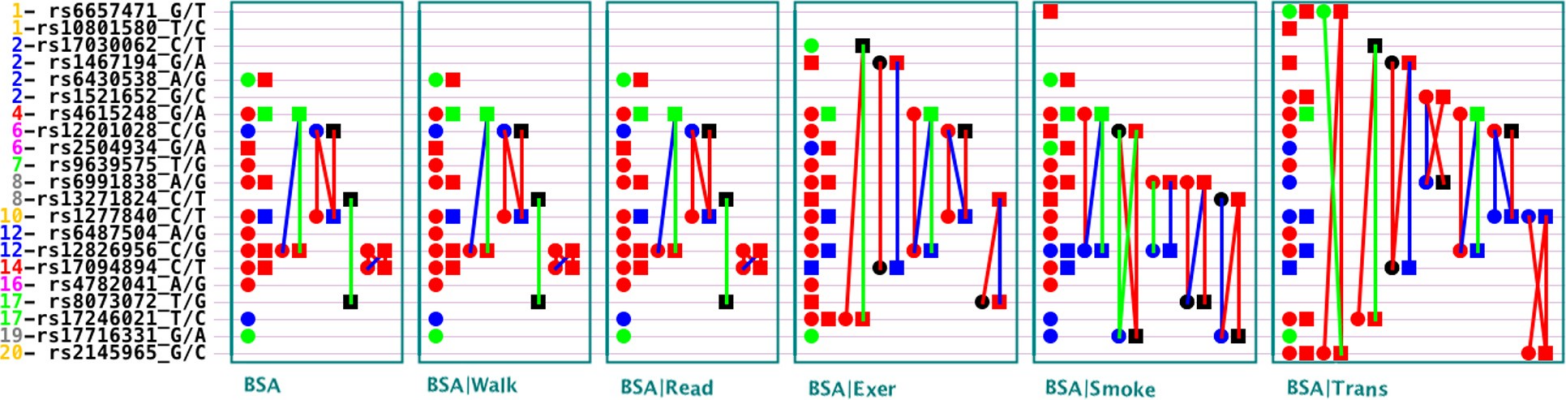
Genetic architecture of detected QTSs for Body Surface Area (BSA) in both base model and five cofactor models with experiment-wise significance (−*Log*_10_ *P*_*EW*_ > 5). BSA: base model; BSA|Walk = walk cofactor model; BSA|Exer = exercise cofactor model; BSA|Read = read cofactor model; BSA|Smoke = smoke cofactor model; and BSA|Trans = transportation cofactor model. Note: The left axis is the QTS IDs: Chromosome-SNP-Alleles; Red circle dot: QTS with additive effects; Green circle dot: QTS with ethnic-specific additive effects; Blue circle dot: QTS with both additive and ethnic-specific additive effects; Red square dot: QTS with dominance effects; Green square dot: QTS with ethnic-specific dominance effects; Blue square dot: QTS with both dominance and ethnic-specific dominance effects; Line between two QTSs = epistasis effects; Red color = QTS with general effects for two race groups; Green color = QTS with ethnicity-specific effects; Blue color = QTS with both general and ethnicity-specific effects; Black color = QTS with ethnicity-specific effects but without detected individual effects.

Although the genetic architecture of complex trait consists of actions of genes in a single locus, inter-locus interactions, and gene-environment interactions [26], most GWASs for obesity-related traits were conducted by ignoring these effects [27], which could largely influence estimated heritability and genetic effects estimation [28]. In this study, epistasis and ethnic-specific genetic effects were revealed as important variants for BSA. From GG plot (Fig 1), it can be observed that the pattern of the genetic architecture of BSA was similar in the base model, and two cofactor models (walk and read). Exercise, smoking, and transportation cofactor models discovered different patterns of genetic architecture. And the differences were mostly for epistasis loci. Several epistasis loci were additionally identified in these cofactors models as well as some base model identified epistasis loci were not identified. Therefore epistasis effects could be very sensitive to lifestyles. We also can observe the genetic pattern in S3 Fig for genetic effects. This figure also suggests that genetic effects could increase or decrease by the different lifestyles.

Fifteen SNPs were highly significant (Experiment-wise *P*_*EW*_ < 1×10^−5^) in the base model, of which thirteen SNPs had individual genetic effects and seven SNPs were involved in four pairs of epistasis interactions. Single SNPs nearby/within five genes (*COL25A1*, *CREB5*, *LMNTD1*, *RP11-81H3*.*2*, *NKG7*) and one pair of epistasis SNPs with the genes *COL25A1* and *RP11-81H3*.*2* were significantly associated with BSA in the base and all the cofactor models (Tables 2 and S2–S5). SNP within the gene *COL25A1* was identified with highly significant additive (*a*≙ –0.017 for G/G, 0.017 for A/A, −*Log*_10_ *P*_*EW*_ = 12.514) and ethnic-specific dominant effects (*de*_*i*_≙ –0.047 for E-A, 0.053 for A-A, and 0.048 for H-A, −*Log*_10_ *P*_*EW*_ = 33.528, 23.887, and 17.587). Race-specific dominance effects of this SNP account for about 2.08% of phenotypic variations. H-A specific dominance effect of this gene increased after removing the effect of smoking. Gene *COL25A1* is brain-specific membrane-bound collagen, containing extracellular collagen domains that associate with senile plaques in Alzheimer disease [29]. Overweight or larger BSA at high ages is a risk factor for dementia, particularly Alzheimer disease [30].

**Table 2:** Predicted genetic effects of individual and epistasis loci with standard error, significance, and heritability for body surface area in the base model.

Chr_SNP_Allele	Gene	Effect	Estimate	SE	–*Log*_10_*P*_*EW*_	*h*^2^(%)	Walk	Exer	Read	Smoke	Trans
2_rs6430538_A/G	*AC016725*.*4*	*d*	0.015	0.003	6.242	0.20	×	√	×	×	√
		*ae*_1_	–0.027	0.004	13.102	1.01	×	√	×	×	√
		*ae*_3_	0.021	0.004	6.960		×	√	×	×	√
4_rs4615248_G/A	*COL25A1*	*a*	–0.017	0.002	12.514	0.47	×	×	×	×	×
		*de*_1_	–0.047	0.004	33.528	2.08	×	×	×	×	×
		*de*_3_	0.053	0.005	23.887		×	×	×	×	×
		*de*_4_	0.048	0.005	17.587		×	×	×	+	×
6_rs12201028_C/G	*RP11–307P5*.*1*	*a*	–0.022	0.002	31.032	0.84	×	+	×	√	×
		*ae*_4_	–0.017	0.004	5.095	0.51	×	√	×	√	√
6_rs2504934_G/A	*SLC22A3*	*d*	0.016	0.003	6.026	0.23	×	×	×	×	√
7_rs9639575_T/G	*CREB5*	*a*	–0.013	0.002	8.240	0.27	×	×	×	×	×
8_rs6991838_A/G	*LINC01299*	*a*	0.01	0.002	5.561	0.18	×	×	×	+	+
		*d*	0.014	0.003	6.427	0.18	×	×	×	–	√
10_rs1277840_C/T	*CACNB2*	*a*	–0.034	0.002	47.164	1.94	×	×	×	×	+
		*d*	0.045	0.003	64.773	1.72	×	–	×	√	–
		*de*_1_	0.026	0.004	10.438	0.51	×	×	×	√	+
		*de*_3_	–0.024	0.005	5.330		√	×	√	√	√
12_rs6487504_A/G	*5*.*8kb 5’ of LMNTD1*	*a*	0.015	0.002	12.423	0.38	×	×	×	×	×
12_rs12826956_C/G	*39kb 5’ of RP11–81H3*.*2*	*a*	–0.026	0.002	36.367	1.14	×	+	×	+	+
		*d*	–0.03	0.003	19.038	0.79	×	+	×	×	+
14_rs17094894_C/T	*54kb 3’ of RP11–907D1*.*1*	*a*	–0.013	0.002	10.806	0.27	–	√	–	×	√
		*d*	–0.031	0.005	9.544	0.82	×	×	×	×	+
16_rs4782041_A/G	*GRIN2A*	*a*	0.012	0.002	7.185	0.23	×	×	×	×	√
17_rs17246021_T/C	*AC005152*.*1*	*a*	0.026	0.002	41.974	1.12	×	–	×	×	×
		*ae*_1_	0.044	0.003	56.241	1.58	×	√	×	×	√
		*ae*_4_	–0.033	0.004	13.296		×	√	×	×	√
19_rs17716331_G/A	*3*.*3kb 5’ of NKG7*	*ae*_1_	–0.016	0.003	5.806	0.33	×	×	×	–	×
4_rs4615248_G/A×12_rs12826956_C/G	*COL25A1×39kb 5’ of RP11–81H3*.*2*	*da*	–0.026	0.003	15.636	1.17	×	×	×	×	×
		*dae*_1_	0.064	0.005	40.822	3.24	×	×	×	×	×
		*dae*_3_	–0.035	0.006	8.357		×	×	×	×	×
		*dde*_1_	0.05	0.006	14.272	1.11	×	×	×	×	×
6_rs12201028_C/G×10_rs1277840_C/T	*RP11–307P5*.*1×CACNB2*	*aa*	0.021	0.003	15.997	1.48	×	×	×	√	×
		*ad*	–0.044	0.003	51.699	3.30	×	+	×	√	×
		*dd*	–0.042	0.006	10.529	1.49	×	×	×	√	×
8_rs13271824_C/T×17_rs8073072_T/G	*13kb 3’ of RP11–785H20*.*1×24kb 3’ of RNF135*	*dde*_4_	0.078	0.015	6.584	5.22	×	×	×	√	√
12_rs12826956_C/G×14_rs17094894_C/T	*39kb 5’ of RP11–81H3*.*2×54kb 3’ of RP11–907D1*.*1*	*aa*	0.01	0.002	5.305	0.34	×	√	×	√	√
		*ad*	0.053	0.005	22.432	4.78	×	√	×	√	√
		*da*	0.021	0.004	8.750	0.75	×	√	×	√	√
		*dd*	0.066	0.013	6.801	3.72	×	√	×	√	√
		*dae*_1_	–0.046	0.005	23.322	2.59	×	√	×	√	√

Genetic effects: *a* = additive effect, *d* = dominance effect, *aa* = additive-additive epistasis effect, *da* = dominance-additive epistasis effect, *aae*_1_ = E-A specific additive-additive epistasis effect, *aae*_2_ = C-A specific additive-additive epistasis effect, *aae*_3_ = A-A specific additive-additive epistasis effect, *aae*_4_ = H-A specific additive-additive epistasis effect, *dae*_1_ = E-A specific dominance-additive epistasis effect, *dae*_2_ = C-A specific dominance-additive epistasis effect, *dae*_3_ = A-A specific dominance-additive epistasis effect, and *dae*_4_ = H-A specific dominance-additive epistasis effect;–*Log*_10_*P*_*EW*_ = minus *log*_10_ (experimental-wise *P*-value), *h*^2^ (%) = heritability (%). Cofactor: walk, exercise, read, smoke, and transportation. Impacts of a cofactor on BSA: “+” is increase BSA, “–” is decrease BSA, “×” is not affected in the cofactor model, “√” is caused/contributed by cofactor.

SNP within the gene *CREB5* had a very significant additive effect (*a*≙ –0.013 for T/T and 0.013 for G/G, −*Log*_10_ *P*_*EW*_ = 8.240), which did not change in the cofactor models. Therefore, the effect of the gene is not affected by lifestyle. *CREB5* gene affects the survival of patients by regulating colorectal cancer metastasis-associated signaling pathways [31]. Moreover, the additive effect of another gene *LMNTD1* also did not significantly changed in the cofactor models. Insertion mutation in *LMNTD1* gene was found in lung cancer patient [32]. Additive and dominance effects of the SNP, which is 39kb 5’ variant of *RP11-81H3*.*2* gene, were significantly larger in exercise and transportation cofactor models as compared to the base model. Therefore, the genetic effect of heterozygote genotype of this variant can be increased for people who do not exercise and do not use excessive transportation. From S4 and S6 Tables, we observed that the reverse case could happen for the peoples of E-A ethnic group who carry heterozygote genotype. If they do not use exercise and do not use transportation, genetic effects of this SNP could be decreased (*d*+*de*_1_≙ –0.03 for the base model, and –0.053 after removing the effect of exercise or transportation). Moreover, for people with homozygous genotypes (C/C or G/G) in this variant, the impact of lifestyle on additive genetic effects may depend on genotypes. Smoking cofactor model identified A-A specific additive effect and E-A specific dominance effect of this SNP (S5 Table). Like exercise and transportation, the impact of smoking on the effect of this SNP may depend on genotypes in the A-A and E-A ethnic groups. Highly significant E-A ethnic specific additive effect (*ae*_1_≙ –0.016 for G/G and 0.016 for A/A, −*Log*_10_ *P*_*EW*_ = 5.806) of SNP in the *NKG7* gene was decreased after removing the effect of smoking. Moreover, smoking cofactor model identified additive effect of this SNP. Epistasis between SNPs of the genes *COL25A1* and *RP11-81H3*.*2* were highly significant for dominance related epistasis effects. This epistasis interaction effects contributed to around 5.52% phenotypic variations, mostly due to ethnic-specific dominance × additive epistasis effects (3.24%). Remarkably, the genetic effects of this epistasis pair did not change in the cofactor models.

### Genetic effects of several SNPs caused by lifestyle cofactors

It was observed that some of the SNPs identified by the base model were not identified in cofactor models. The genetic effects of these SNPs may be caused by the respective cofactors. From Table 2, it can be observed that one or some of the lifestyle cofactor models did not identify genetic effects of several single and epistasis SNPs.

For example, the genetic additive effect (*a*≙ 0.012 for A/A and –0.012 for G/G, −*Log*_10_ *P*_*EW*_ = 7.185) of SNP of the gene *GRIN2A* and dominance effect (*d*≙ 0.016 for G/A, −*Log*_10_ *P*_*EW*_ = 6.026) of SNP of the gene *SLC22A3* were not highly significantly identified using transportation cofactor model, indicating that transportation associate with genetic effects of SNPs of these genes. The role of these SNPs could be either positive or negative depending on its genotypes, indicating that transportation could have both positive and negative impacts on BSA due to SNPs of these genes. If people carry A/A genotype of SNP of the gene *GRIN2A* and G/A genotype of SNP of the gene *SLC22A3*, the impact of transportation may be positive on BSA. If people carry G/G genotype of SNP of *GRIN2A* and any homozygous genotypes of SNP of *SLC22A3*, then the impact of transportation may be negative on BSA. Because sum of the genetic effects of two genes will be positive for A/A genotype of SNP of gene *GRIN2A* and G/A genotype of SNP of gene *SLC22A3* and will be negative for G/G genotype of SNP of *GRIN2A* and any homozygous genotypes of SNP of *SLC22A3*. Transportation and exercise associate with dominance and ethnic-specific additive effects (E-A and A-A specific) of SNP of the gene *AC016725*.*4*, the additive effect of SNP of gene *RP11–907D1*.*1*, and ethnic-specific additive effects (E-A and H-A specific) of SNP of the gene *AC005152*.*1*. These results indicate that the genetic effects of these genes depend on both exercising and use of transportation. Similarly, H-A ethnic-specific additive effect of SNP of the gene *RP11-307P5*.*1*, epistasis effects (additive × additive, additive × dominance, dominance × additive, dominance × dominance, and E-A specific dominance × additive) of SNPs of the genes *RP11-81H3*.*2* and *RP11-907D1*.*1* were associated with transportation, exercise, and smoking. SNPs of the genes *RP11-307P5*.*1* and *CACNB2* had highly significant additive × additive (0.021 for C/C × C/C and G/G × T/T, and –0.021 for C/C × T/T and G/G × C/C), additive × dominance (–0.044 for C/C × C/T and 0.044 for G/G × C/T), and dominance × dominance (–0.042 for C/G × C/T) interaction effects in the base model. However, the effects of the SNPs of these epistasis genes were not identified by using smoking cofactor model.

Genetic effects of base model identified several genes were significantly increased or decreased in the lifestyle cofactor models (Table 2). For example, additive (*a*≙ 0.0102 for A/A and –0.0102 for G/G, −*Log*_10_ *P*_*EW*_ = 5.561) and dominance (*d*≙ 0.0144 for A/G, −*Log*_10_ *P*_*EW*_ = 6.427) effects were detected for a SNP of gene *LINC01299* in the base model. The additive effect of this gene increased in cofactor models of transportation and smoking. The dominance effect was not detected in transportation cofactor model but decreased in smoking cofactor model. These results indicated that lifestyle cofactors could increase or decrease genetic effects of several genes.

### Genetic effects of several SNPs suppressed by lifestyle cofactors

SNPs of seven single and eight pairs of epistasis genes were highly significant only in the cofactor models (listed in Table 3).

**Table 3:** Predicted genetic effects of individual and epistasis loci with standard error, significance, and heritability for BSA associated loci identified only in the lifestyle cofactor models.

Model	Chr_SNP_Allele	Gene	Effect	Estimate	SE	–*Log*_10_*P*_*EW*_	*h*^2^(%)
**BSA|Exer**	2_rs17030062_C/T	*ACTR2*	*ae*_3_	0.024	0.004	10.423	0.46
	2_rs1467194_G/A	*TMEM163*	*d*	0.012	0.003	5.126	0.11
	8_rs13271824_C/T	*13kb 3’ of RP11–785H20*.*1*	*d*	–0.038	0.004	22.866	1.12
	2_rs17030062_C/T×17_rs17246021_T/C	*ACTR2×AC005152*.*1*	*da*	–0.041	0.006	12.208	2.52
			*dde*_4_	–0.083	0.010	16.014	5.22
	2_rs1467194_G/A×14_rs17094894_C/T	*TMEM163×54kb 3’ of RP11–907D1*.*1*	*aa*	0.024	0.002	22.795	1.76
			*dd*	–0.044	0.008	8.235	1.44
			*dde*_4_	0.047	0.010	5.738	1.30
	8_rs13271824_C/T×17_rs8073072_T/G	*13kb 3’ of RP11–785H20*.*1×24kb 3’ of RNF135*	*da*	0.030	0.004	13.207	1.38
			*dd*	–0.116	0.013	19.631	10.30
			*dde*_4_	0.085	0.015	7.734	5.55
**BSA|Smoke**	1_rs6657471_G/T	*9*.*1kb 3’ of RP4–771M4*.*3*	*d*	0.013	0.003	5.454	0.14
	8_rs13271824_C/T	*13kb 3’ of RP11–785H20*.*1*	*d*	0.040	0.004	24.973	1.27
	8_rs6991838_A/G×12_rs12826956_C/G	*CTD-3025N20*.*2×39kb 5’ of RP11-81H3*.*2*	*dd*	0.039	0.005	13.171	1.22
			*aae*_*3*_	–0.023	0.005	6.077	0.85
			*dde*_1_	–0.032	0.007	6.115	1.87
	8_rs6991838_A/G×17_rs8073072_T/G	*CTD–3025N20*.*2×24kb 3’ of RNF135*	*aa*	–0.019	0.002	15.599	1.19
			*da*	0.028	0.003	19.05	1.21
			*dd*	0.037	0.008	5.592	1.06
			*dae*_1_	0.023	0.004	8.611	1.05
	8_rs13271824_C/T×19_rs17716331_G/A	*13kb 3’ of RP11–785H20*.*1×3*.*3kb 5’ of NKG7*	*aa*	–0.038	0.002	54.928	4.68
			*da*	–0.058	0.004	38.417	5.35
			*dd*	–0.076	0.008	22.913	4.53
			*aae*_3_	0.064	0.005	40.255	4.33
			*aae*_4_	–0.030	0.006	7.141	
**BSA|Trans**	1_rs6657471_G/T	*9*.*1kb 3’ of RP4–771M4*.*3*	*d*	0.025	0.003	17.585	0.44
			*ae*_1_	0.020	0.003	8.547	0.37
	1_rs10801580_T/C	*CFHR2*	*d*	–0.013	0.003	5.142	0.12
	2_rs1467194_G/A	*TMEM163*	*d*	0.014	0.003	7.102	0.14
	2_rs1521652_G/C	*ERBB4*	*a*	–0.018	0.002	19.737	0.47
			*d*	–0.038	0.004	27.734	1.04
	20_rs2145965_G/C	*29kb 5’ of RP5–1177M21*.*1*	*a*	–0.010	0.002	5.012	0.14
			*d*	0.016	0.003	8.978	0.18
	1_rs6657471_G/T×20_rs2145965_G/C	*9*.*1kb 3’ of RP4–771M4*.*3×29kb 5’ of RP5–1177M21*.*1*	*da*	–0.018	0.004	5.549	0.47
			*dd*	–0.026	0.004	8.711	0.46
			*ade*_1_	–0.023	0.005	5.749	0.56
	2_rs17030062_C/T×17_rs17246021_T/C	*ACTR2× AC005152*.*1*	*da*	–0.045	0.006	14.73	2.79
			*dde*_4_	–0.088	0.010	18.375	8.30
	2_rs1467194_G/A×14_rs17094894_C/T	*TMEM163×54kb 3’ of RP11–907D1*.*1*	*aa*	0.022	0.002	19.297	1.34
			*da*	–0.014	0.003	6.007	0.27
			*dd*	–0.041	0.007	7.42	1.16
			*dde*_4_	0.053	0.010	7.259	1.43
	2_rs1521652_G/C×8_rs6991838_A/G	*ERBB4×CTD–3025N20*.*2*	*aa*	–0.027	0.003	27.342	2.10
			*ad*	0.023	0.003	12.094	0.74
			*da*	–0.027	0.004	9.083	1.00
			*aae*_1_	–0.024	0.004	8.428	2.70
			*aae*_4_	0.037	0.006	9.09	
	10_rs1277840_C/T×20_rs2145965_G/C	*CACNB2×29kb 5’ of RP5–1177M21*.*1*	*ad*	–0.024	0.004	10.504	0.81
			*da*	0.017	0.004	5.545	0.38
			*dd*	0.022	0.004	8.043	0.33

**Genetic effects:** *a* = additive effect, *d* = dominance effect, *aa* = additive-additive epistasis effect, *da* = dominance-additive epistasis effect, *aae*_1_ = E-A specific additive-additive epistasis effect, *aae*_2_ = C-A specific additive-additive epistasis effect, *aae*_3_ = A-A specific additive-additive epistasis effect, *aae*_4_ = H-A specific additive-additive epistasis effect, *dae*_1_ = E-A specific dominance-additive epistasis effect, *dae*_2_ = C-A specific dominance-additive epistasis effect, *dae*_3_ = A-A specific dominance-additive epistasis effect, and *dae*_4_ = H-A specific dominance-additive epistasis effect;–*Log*_10_*P*_*EW*_ = minus *Log*_10_ (experimental-wise *P*-value), *h*^2^ (%) = heritability (%). Cofactor: exercise, smoke and transportation.

For the identified SNPs of thirteen genes, none of the genetic variants of SNPs of seven genes (*RP4-771M4*.*3*, *CFHR2*, *ERBB4*, *ACTR2*, *TMEM163*, *CTD-3025N20*.*2*, and *RP5-1177M21*.*1*) were identified using the base model. Transportation cofactor model identified effects of SNPs of five single genes (*RP4-771M4*.*3*, *CFHR2*, *TMEM163*, *ERBB4*, and *RP5-1177M21*.*1*) and five pairs of epistasis genes (*RP4-771M4*.*3* × *RP5-1177M21*.*1*, *ACTR2* × *AC005152*.*1*, *TMEM163* × *RP11-907D1*.*1*, *ERBB4* × *CTD-3025N20*.*2*, and *CACNB2* × *RP5-1177M21*.*1*). For example, SNP of gene *ERBB4* was identified using only transportation cofactor model with additive (*a*≙ –0.018 for G/G and 0.018 for C/C, −*Log*_10_ *P*_*EW*_ = 19.737) and dominance (*d*≙ –0.038 for G/C, −*Log*_10_ *P*_*EW*_ = 27.734) effects. Gene *ERBB4* encodes receptor tyrosine-protein kinase erbB-4 that plays an essential role as a cell surface receptor for neuregulins. This gene had been identified as a potential risk gene for bipolar disorder and schizophrenia [33,34]. SNP of the gene *ERBB4* is also involve in epistasis interaction with the SNP of gene *CTD-3025N20*.*2* in transportation cofactor model, with highly significant additive × additive (*aa*≙ –0.027 for G/G × A/A and C/C × G/G, 0.027 for G/G × G/G and C/C × A/A, −*Log*_10_ *P*_*EW*_ = 27.342), additive × dominance (*ad*≙ 0.023 for G/G × A/G, and –0.023 for C/C × A/G, −*Log*_10_ *P*_*EW*_ = 12.094), dominance × additive (*da*≙ –0.027 for G/C × A/A and 0.027 for G/C × G/G, −*Log*_10_ *P*_*EW*_ = 9.083), E*-*A and H*-*A ethnic-specific additive × additive (*aae*_1_≙ –0.024 for G/G × A/A and C/C × G/G, 0.024 for G/G × G/G and C/C × A/A, −*Log*_10_ *P*_*EW*_ = 8.428, and *aae*_4_≙ 0.037 for G/G × A/A and C/C × G/G, –0.037 for G/G × G/G and C/C × A/A, −*Log*_10_ *P*_*EW*_ = 9.09) epistasis effects. Exercise cofactor model newly identified genetic effects of SNPs of three single genes (*ACTR2*, *TMEM163*, and *RP11-785H20*.*1*) and three pairs of epistasis genes (*ACTR2* × *AC005152*.*1*, *TMEM163* × *RP11-907D1*.*1*, and *RP11-785H20*.*1* × *RNF135*). Smoking cofactor model newly identified genetic effects of SNPs of two single genes (*RP4-771M4*.*3*, and *RP11-785H20*.*1*) and three pairs of epistasis genes (*CTD-3025N20*.*2* × *RP11-81H3*.*2*, *CTD-3025N20*.*2* × *RNF135* and *RP11-785H20*.*1* × *NKG7*). Epistasis effects between SNPs of genes *RP11-785H20*.*1* and *RNF135* were identified in exercise and smoking cofactor models, with some additional genetic variants as compared to the base model. Since the effects of SNPs of these identified genes depend on their genotypes and ethnicity of individual observations, therefore impacts of lifestyle on BSA may also be varied in different ethnic groups.

### Genetic effects of the several SNPs differs for the peoples of different ethnic population

**Fig 2:**
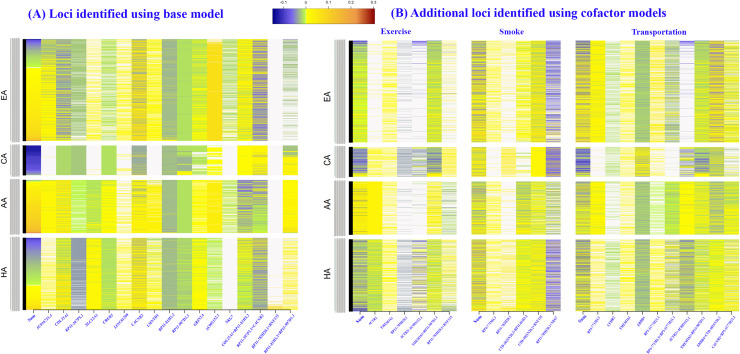
Genetic effects matrix (G + GE) image plot of BSA loci. (A) Identified loci using the base model; and (B) suppressed loci by cofactors. Vertical axis for the size of genetic effects including four ethnic groups: HA = Hispanic-American, AA = African-American, CA = Chinese-American, EA = European-American; horizontal axis for the individual and epistasis loci; different color present different genetic effects according to a color scale, where gray color = no significant effects.

Genetic effects for four different ethnic groups were estimated using the base model and plotted in Fig 2A. It was observed that each ethnic group is genetically different from other ethnic groups based on genetic effects of BSA associated genes.

Percentages of individuals carrying positive and negative effects corresponding to the identified loci using the base model were tabulated in S7 Table. It was observed that for epistasis between SNPs of genes *RP11-785H20*.*1* and *RNF135*, peoples of E-A, C-A, and A-A ethnic groups had no genetic effects, but 5.32% peoples of H-A groups had significant positive effects who carry C/T × T/G genotypes. Corresponding to the SNP of gene *NKG7*, all the peoples of C-A and H-A groups had no significant genetic effects, but 9.32% peoples had positive effects (0.016) for A/A genotype and 51.47% peoples had negative effects (–0.016) for G/G genotype in E-A ethnic groups. Moreover, for A-A ethnic groups 9.62% peoples had a positive effect (0.011) for G/G genotype and 49.52% peoples had negative effects (–0.011) for A/A genotype. Therefore, the same genotype of a SNP could have completely different genetic effect in different ethnic groups. For 5.8kb 5’ variant of *LMNTD1*, in C-A groups 49.26% peoples had negative effects and 41.39% peoples had no effects, but 72.19% of E-A individuals and 82.68% of A-A individuals had positive effects.

In E-A ethnic group, 69.96 ~ 96.36% of peoples had positive effects for SNPs of four genes (*AC016725*.*4*, *CACNB2*, *LMNTD1*, and *AC005152*.*1*), and one pair of epistasis genes *COL25A1* × *RP11–81H3*.*2*; but 56.34 ~ 94.42% peoples had negative effects for SNPs of four genes (*CREB5*, *COL25A1*, *RP11–307P5*.*1*, and *RP11–81H3*.*2*), and one pair of epistasis genes *RP11–307P5*.*1* × *CACNB2*. It was observed that 65.73 ~ 99.55% of C-A peoples had negative effects corresponding to the SNPs of seven genes (*COL25A1*, *RP11–307P5*.*1*, *CREB5*, *CACNB2*, *RP11–81H3*.*2*, *RP11–907D1*.*1*, and *GRIN2A*); but 91.54 ~ 98.96% peoples had positive effects corresponding to SNPs of two pairs of epistasis genes (*COL25A1* × *RP11–81H3*.*2* and *RP11–307P5*.*1* × *CACNB2*). Moreover, 98.96 ~ 100% C-A peoples had no significant genetic effects corresponding to SNPs of four genes (*AC016725*.*4*, *SLC22A3*, *LINC01299*, and *NKG7*) and one pair of epistasis genes *RP11–785H20*.*1* × *RNF135*. From S9 Table, it can be observed that Chinese-Americans have a lower death rate in breast cancer, may be due to negative or no effects of *LMNTD1* gene, and the negative effect of *CREB5* gene.

In A-A ethnic group, 82.45 ~ 97.92% peoples had positive effects corresponding to SNPs of seven genes (*AC016725*.*4*, *COL25A1*, *CREB5*, *CACNB2*, *LMNTD1*, *GRIN2A*, and *AC005152*.*1*) and one pair of epistasis genes (*RP11–81H3*.*2* × *RP11–907D1*.*1*), but 75.24 ~ 100% peoples had negative effects corresponding to SNPs of four genes (*RP11–307P5*.*1*, *SLC22A3*, *RP11–81H3*.*2*, and *RP11–907D1*.*1*) and two pairs of epistasis genes (*COL25A1* × *RP11–81H3*.*2*, and *RP11–307P5*.*1* × *CACNB2*).

In H-A ethnic group, 75.61 ~ 92.27% peoples had positive effects corresponding to SNPs of three genes (*SLC22A3*, *CACNB2*, and *GRIN2A*), and 75.53 ~ 97.87% peoples had negative effects corresponding to SNPs of four genes (*RP11–307P5*.*1*, *CREB5*, *RP11–81H3*.*2*, and *RP11–907D1*.*1*), and one pair of epistasis genes (*RP11–307P5*.*1* × *CACNB2*). It is noted that incidence and death rate in lung cancer is lowest in the Hispanic-American ethnic group, which could be due to a higher frequency of negative effect alleles of SNPs of *CREB5* gene in this ethnic group.

Corresponding to SNPs of three genes (*RP11–307P5*.*1*, *RP11–81H3*.*2*, and *RP11–907D1*.*1*), 75.24 ~ 99.55% peoples of four ethnic groups had negative effects. Interestingly, individuals in C-A ethnic group carry negative effects and no effects respect to more of the genes as compared to other three ethnic groups. In converse, peoples of A-A ethnic groups carry positive effects with respect to more genes. From phenotypic distribution in the MESA population, plotted in S1 Fig, it can be observed that average BSA was lower for C-A ethnic groups but larger for A-A ethnic groups.

From S4A Fig, we observed that average of total genetic effects differ among ethnic groups, smaller for C-A individuals but larger for A-A individuals. For E-A ethnic group, 72.23% individuals had positive total genetic effects, but C-A ethnic group had negative total genetic effects, 96.31% A-A ethnic group had positive genetic effects, and 65.55% H-A ethnic group had negative total genetic effects. An average total genetic effect was smaller for C-A individuals. Moreover, from S4B Fig we observed an average of G×E effects was also smaller for C-A individuals. Therefore, the average BSA was smaller for C-A individuals due to both genetic and G × E effects.

Exercise, smoking, and transportation cofactor models identified several single and epistasis genes that were not detected by the base model (S2B Fig). We observed the different pattern of genetic effects of SNPs of these genes in different ethnic groups. Percentages of individuals carrying positive and negative effects corresponding to SNPs of the additional genes identified using cofactor models were listed in S8 Table. It was observed that exercise cofactor model identified effects of a SNPs of gene *ACTR2*, for which 100% A-A peoples had positive effects, but 97.63% E-A peoples had no effects, and 65.71% H-A peoples had negative effects. For the SNPs of gene *TMEM163*, 32.45 ~ 48.85% peoples had positive effects across ethnic groups. Exercise cofactor model identified SNPs of three single and three pairs of epistasis genes. Among them, SNPs of two genes (*ACTR2* and *TMEM163*) and one pair of epistasis genes (*RP11–785H20*.*1* × *RNF135*) had positive effects for 35.31 ~ 51.78% C-A individuals. Moreover, SNP of gene *RP11–785H20*.*1*, and SNPs of two pairs of epistasis genes (*ACTR2* × *AC005152*.*1* and *TMEM163* × *RP11–907D1*.*1*) had negative effects for 52.08 ~ 82.05% individuals. Therefore, removing exercising may have negative impacts on BSA for many C-A individuals. However, SNPs of two single genes (*ACTR2* and *TMEM163*) and one pair of epistasis genes (*TMEM163* × *RP11–907D1*.*1*) had positive effects for 32.45 ~ 100% A-A individuals, whereas SNPs of two pairs of epistasis genes (*TMEM163* × *RP11–907D1*.*1* and *RP11–785H20*.*1* × *RNF135*) had negative effects for 38.06 ~ 40.14% A-A individuals. Therefore, removing exercising may have positive impacts on BSA for many A-A individuals.

Smoking cofactor model identified SNPs of two single genes and three pairs of epistasis genes, whereas SNPs of two genes (*RP4–771M4*.*3* and *RP11–785H20*.*1*) had positive effects for 32.32 ~ 37.97% H-A individuals and SNPs of two pairs of epistasis genes (*CTD–3025M20*.*2* × *RP11–81H3*.*2* and *CTD–3025M20*.*2* × *RNF135*) had positive effects for 70.13 ~ 74.96% H-A individuals. Therefore, removing smoking may have a positive impact on BSA for many H-A individuals. Similar results also found for E-A and C-A ethnic groups. Transportation cofactor model identified five single and five pairs of epistasis genes. SNP of gene *ERBB4* had positive effects for 61.13% C-A individuals, but negative effects for 92.06 ~ 99.28% individuals of other ethnic groups. SNP of gene *RP4–771M4*.*3* had a positive effect for more than 90% individuals of E-A and A-A ethnic groups.

### Gene ontology analysis

**Fig 3:**
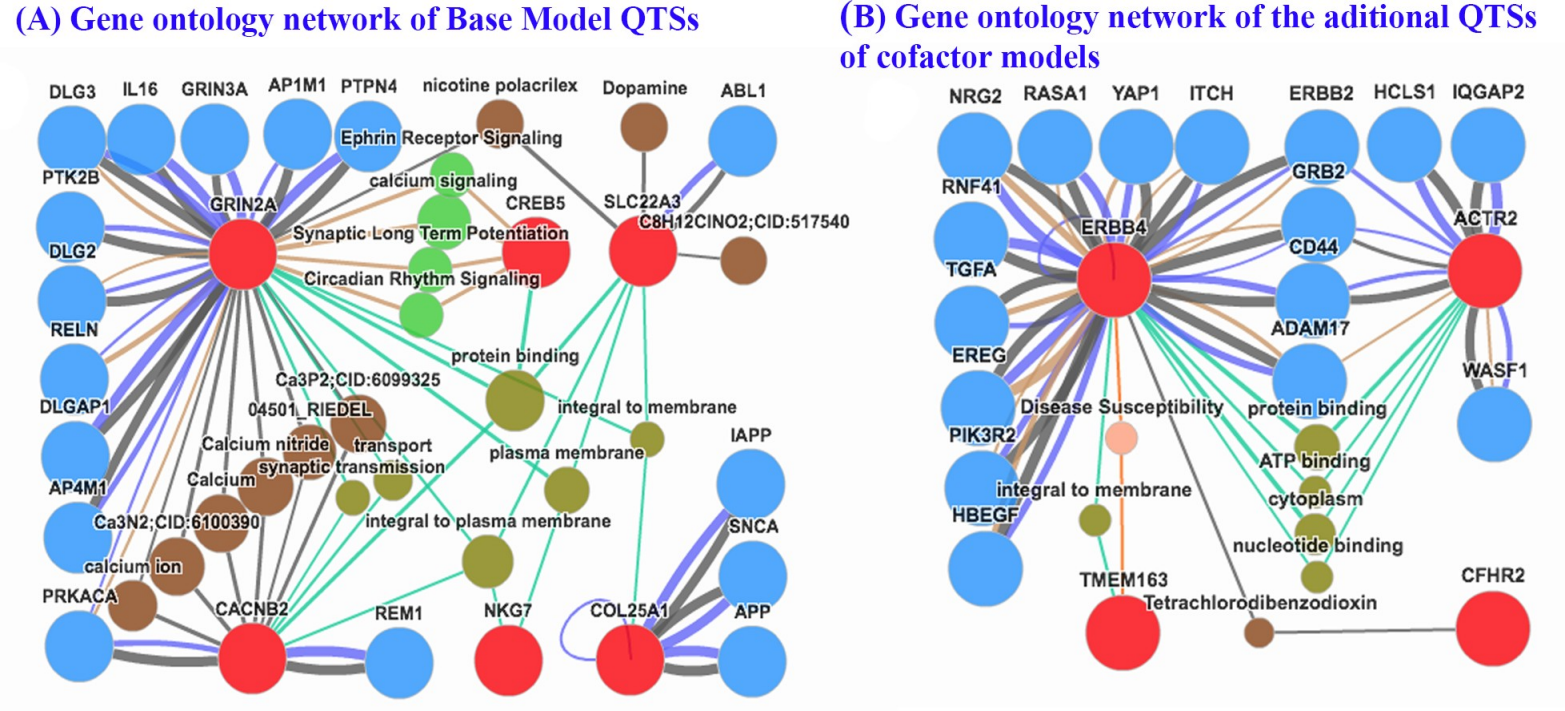
Genetic architecture of detected quantitative trait signal nucleotide polymorphism (QTSs) for BSA. (A) QTSs detected in both the BSA symptom count (non-cofactor) model and individual cofactor models with high significance (–*Log*_10_*P*_*EW*_ > 5); (B) QTSs detected for BSA symptom count only in a suppressed model with high significance (–*Log*_10_*P*_*EW*_ > 5). The size of balls and thickness of lines stand for a number of publications related. Red balls represent seed genes detected; orange balls represent association diseases; Olive balls represent association functions; Brown balls represent association chemicals; Royal blue balls represent association genes. Red-orange lines represent gene-disease association; Paris green lines represent gene ontology; Dark blue lines represent protein-protein interaction; Bronze lines represent pathway interaction; Dark gray lines represent the database.

Using ***Biopubinfo*** (http://ibi.zju.edu.cn/biopubinfo/), the structural and functional connectivity among highly significant SNPs (*P*_EW_ < 1×10^−5^) within or near candidate genes were evaluated. The candidate genes corresponding to detected SNPs were used as seeds. The networks among candidate genes, related pathway, functions, genes, chemical and drug information, protein-protein interactions, and the gene-disease association was generated using ***Biopubinfo*** and shown in Fig 3.

Networks were constructed separately for the candidate genes from the base model (Fig 3A) and additional genes from cofactor models (Fig 3B). We observed that two candidate genes *GRIN2A* and *CACNB2* have a relationship with several common chemicals including calcium, calcium ion, calcium nitride, and other calcium-related chemicals (Fig 3A). Both genes are involved in protein-protein interaction with gene *PRKACA* and have common function transportation. BSA is a predictor of coronary artery calcium (CAC). An elevated level of BSA is associated with CAC incidence [7]. The CAC score is the amount of calcium in the walls of the arteries. Genes *GRIN2A* and *SLC22A3* have relationship with chemical nicotine polacrilex that is used for nicotine replacement therapy in smoking cessation. Gene *SLC22A3* also has a relationship with dopamine and dopamine hydrobromide chemicals. Dopamine (DA, a contraction of 3, 4-dihydroxyphenethylamine) is an organic chemical that plays several important roles in the brain and body. Previous GWAS analyses showed that the variant of *SLC22A3* gene is associated with coronary artery disease [35].

Two genes *GRNI2A* and *CREB5* displayed association signaling in circadian rhythm signaling, calcium signaling, ephrin receptor signaling, and synaptic long-term potentiation function. There were 4 genes (*GRIN2A*, *CREB5*, *SLC22A3*, and *CACNB2*) involved in protein binding and represent protein-protein interaction. Genes *GRIN2A*, *NKG7*, and *SLC22A3* had functions in Plasma membrane. Four genes (*GRIN2A*, *CACNB2*, *NKG7*, and *SLC22A3*) also represent association functions in integral to the plasma membrane. Gene *COL25A1* connected with the other three genes *APP*, *SNCA*, and *LAPP*. Association of BMI with a variant of *COL25A1* gene was significant in previous GWAS [36]. Therefore, the gene may control the phenotypic variation of BMI and BSA. Variants of *CREB5* and *5*.*8kb 5’ of LMNTD1* had only highly significant additive effects. Gene *LMNTD1* is a protein-coding gene associated with Respiratory System Benign Neoplasm. Previous studies showed that *LMNTD1* gene was related to the average daily gain of beef cattle [37]. *GRIN2A* is a protein-coding gene, and GO annotations related to the gene include calcium channel activity and ionotropic glutamate receptor activity. Previous GWAS identified a significant association of the variant of *LINC01299* with sleeping duration [38], which is a risk factor for obesity, especially for childhood obesity [39,40].

Four candidate genes corresponded to highly significant QTSs were newly identified in the lifestyle cofactor models (Fig 3B). Gene *ERBB4* relates to three genes *ACTR2*, *TMEM163*, and *CFHR2*. There was remarkable connectivity for the genes of *ERBB4* and *ACTR2* based on protein-protein interaction with other genes (*ERBB2*, *GRB2*, *CD44*, and *ADAM17*) and both shared some common ontology (protein binding, ATP binding, nucleotide binding, and cytoplasm). Genes *ERBB4* and *TMEM163* had functions in integral to membrane, and represent association to disease susceptibility. We also observed that two candidate genes *ERBB4* and *CFHR2* have a relationship with common chemical Tetra-chloro-dibenzo-dioxin (TCDD).

## Discussion

Body surface area is a complex trait related to coronary artery calcium (CAC), coronary artery disease (CAD), and cancer. It has been widely used to determine the appropriate dose of the drug [1,5–8]. This is an indicator of normal weight and obesity [41]. Analyses results suggested that BSA was determined by complicated genetic and environmental factors and their interactions. Moreover, lifestyle cofactors largely influence the genetic effects of BSA associated genes. Non-additive and ethnic-specific genetic effects have been largely ignored in GWAS for complex traits of human, animals, and plants [28]. However, several recently published GWAS of human and plant complex traits revealed the importance of non-additive genetic effects and their large contributions to phenotypic variations [28,42]. Importance of non-additive genetic effects also revealed in this study, and these effects largely contribute to the phenotypic variations of BSA. By testing genetic main effects, epistasis effects, as well as effects of genetic by ethnicity, the total estimated heritability of BSA was 74.85%. The contribution of dominance and dominance-related epistasis effects ($h_{D+}^{2}≙$ 58.92%) was high. Moreover, large phenotypic variations were due to ethnic-specific dominance and dominance-related epistasis effects (38.71%), suggested that effects of heterozygous genotypes are important contributors to the phenotypic variation of BSA, and their effects could be largely varied across different ethnic groups. High heritability was also estimated for racial-specific genetic effects in different models, and genetic effects of loci were largely varied across different ethnic groups. Therefore, heterozygote genotypes of several identified loci play important roles in controlling the genetic variation of BSA, and genetic effects significantly vary across different ethnic groups. Although estimated total heritability did not greatly differ due to different lifestyle cofactors, some component heritability was significantly changed. Especially, heritability due to dominance and dominance-related epistasis effects were significantly varied in cofactor models. These results indicated that different types of lifestyle cofactors might have different impacts on genetic effects of the identified SNPs. Therefore, effects of heterozygote genotypes of several loci are very sensitive to lifestyles. Heritability estimation provides indications of influence for lifestyle cofactors on genetic effects. Thus, estimated heritability by using different models was varied due to increasing or decreasing genetic effects of several SNPs by the influence of different lifestyles.

Doctors and physiologists often recommend specific lifestyle control, like exercises and smoking, to achieve better health and skip diseases related to obesity. However, it is unknown that how lifestyle cofactor could control these complex traits. This study suggests that lifestyles influences on genetic underpinnings to do that. It was revealed that lifestyle cofactors could have large influences on genetic effects of BSA associated genes. Lifestyle may increase or decrease genetic effects. Fifteen highly significant BSA associated genes (experiment-wise *P*_EW_ < 1 × 10^−5^) were identified in the base model (unconditional model), including thirteen single genes and four pairs of epistasis genes (Table 2). Moreover, after removing the effects of transportation, exercise, and smoking cofactors (conditional models), seven single genes and eight pairs of epistasis genes were newly identified (Table 3, Figs 1 and S3). However, several genes identified in the base model were not identified in the cofactor models; and if we remove effects of lifestyles then the expression of several genes might be increased, by the same time expression of several other genes might be decreased.

It was revealed that different lifestyle cofactors might have different impacts on genetic effects. Transportation, exercise, and smoking had large impacts on the identified genes as compared to walk and read (Figs 1 and S3). It is expected that transportation, exercise, and smoking could have large influences on BSA. In the base model, it was observed that only effects of SNPs of three individual genes (*COL25A1*, *CREB5*, and *LMNTD1*) and one pair of epistasis genes (*COL25A1* × *RP11-81H3*.*2*) were not affected by all the lifestyle cofactors used in this study. These results demonstrate that lifestyle cofactors may largely influence at the genetic levels for controlling BSA.

The genetic effects observed for individuals depend not only on genotype but also on their ethnicity. Peoples of different ethnic groups generally have different genetic backgrounds across the whole genome and have different lifestyles. It was observed that C-A individuals had lower BSA as compared to other ethnic groups that could be due to genotypic differences of some loci and/or interaction of genetic variants with ethnicity (S1 Fig). From analyses results it was also observed that genetic effects of C-A individuals were different from A-A, E-A, and H-A individuals due to both genotypic differences and ethnicity interactions of some loci (Fig 2). These results suggest that race information is important for designing personalized medicine. Moreover, lifestyles may influence genetic effects of SNPs, which could determine positive or negative impacts of lifestyle on BSA in different ethnic groups. For example, conditional association analyses suggested that genetic effect of heterozygote genotype of the SNP within the gene *COL25A1* could be larger for non-smokers as compared to smokers in the H-A ethnic group. However, there had no such indication for this gene in the case of other ethnic groups. Lifestyle could change the effects of several genes, but not for all associated genes. Moreover, even if BSA can be reduced by setting a specific lifestyle, but that might not helpful for patients of BSA associated diseases. For example, cancer-related genes *CREB5* and *LMNTD1* are associated with BSA, but are not related to the lifestyles used in this study. Therefore, controlling lifestyle exposure used in this study may not be helpful for cancer patients. Moreover, increasing BSA may not always lead to cancer development, but why the increase in BSA may be a major factor. If BSA increases due to the expression of cancer-associated genes, this may be the cause of cancer. Impacts of lifestyle on the genetic effects of loci in the different individuals may depend on genotypes of SNPs they carry. And, although genetic effects could be varied due to a different lifestyle, all the effects of SNPs of several genes (*CREB5*, *LMNTD1*, *COL25A1*, *RP11-81H3*.*2*, and *NKG7*) may not be affected by lifestyle cofactors. For *LMNTD1*, in C-A groups 49.26% peoples had negative effects and 41.39% peoples had no effects, but 72.19% of E-A individuals and 82.68% of A-A individuals had positive effects. For *CREB5*, 99.26% C-A peoples had negative effect, but 88.46% of A-A individuals had positive effects.Remarkably, incidence and death rate of cancer is highest in African-American ethnic group [43] (S9 Table), whereas one of the causes of higher incidence and death rate may be due to the large frequency of positive effect alleles of *CREB5* and *LMNTD1* genes in this ethnic group (S7 Table).

Candidate genes corresponding to the identified SNPs had an association with several traits or diseases, including CAD, CAC, Alzheimer disease, type-2 diabetes, and cancer. For example, *COL25A1* is brain-specific membrane-bound collagen that comprises an extracellular collagen domain associated with senile plaques in Alzheimer’s disease (AD; MIM 104300) [29]. *COL25A1* is specifically expressed in neurons and binds to aggregated αβ in vitro [44]. Genome-Wide Gene-Environment Study identifies Glutamate Receptor Gene *GRIN2A* as a Parkinson’s disease Modifier Gene, which encodes an NMDA-glutamate-receptor subunit and regulates excitatory neurotransmission in the brain [45]. Evidence from epidemiological studies suggests a relationship between cigarette smoking and low risk of Parkinson disease (PD). *SLC22A3* encodes an organic cation transporter with diabetic nephropathy and hypertension with a broader pattern of expression including the small intestine, liver, kidney, placenta, skeletal muscle, heart and brain [46]. The variant of *LINC01299* is associated with sleeping duration [38], which is a risk factor for obesity [39,40]. Bioinformatics analysis using *BiopubInfo* found that three genes *GRIN2A CACNB2*, and *SLC22A3* were associated with calcium and calcium compounds, whereas literature search revealed that BSA is a predictor of coronary calcium. Moreover, *CREB5* and *LMNTD1* genes are associated with cancer.

In this study, we focused on impact of five different lifestyle cofactors. There are more other exposures like diet and alcohol intake might influence BSA associated loci. However, some of these factors were not available for MESA population and other ignored to reduce complexity of the study.

## Supporting information

S1 Fig Box plot for age, sex, and phenotypic distribution. EA = European- American, CA = Chinese-American, AA = African-American, and HA = Hispanic-American.
(PDF)

S2 Fig Bar plot for body surface area and five different cofactor models. Different colors used for indicating heritability due to different types of genetic effects.
(PDF)

S3 Fig Genetic and Genetic by Ethnic interaction effects plot for BSA base model and life-style cofactor models. The size of the vertical axis is the genetic effects, and the horizontal axis is the SNP name and effects type.
(PDF)

S4 Fig Genetic effects matrix (G and GE) image plot of BSA loci. (A) Genetic main effects of loci and (B) G × E effects of loci. The vertical axis for the size of genetic effects including four ethnic groups: HA = Hispanic-American, AA = African-American, CA = Chinese-American, EA = European-American; horizontal axis for the individual and epistasis loci; different color present different genetic effects according to a color scale, where gray color = no significant effects.
(PDF)

S1 Table Allele frequencies of the identified loci. CHR refers chromosome; SNP refers SNP ID; MAF refers minor allele frequency.
(PDF)

S2 Table Predicted genetic effects of individual and epistasis loci with standard error, significance, and heritability for BSA|Walk cofactor model. QTS: identified quantitative trait SNP; Gene: near or holder gene ID; Effect: type of gene effects;–*log*_*10*_*P*_*EW*_: minus log experimental-wise P-value; %: estimated heritability for the effects; Gene Description: description of the candidate genes collected from NCBI gene database.
(PDF)

S3 Table Predicted genetic effects of individual and epistasis loci with standard error, significance, and heritability for BSA|Read cofactor model. QTS: identified quantitative trait SNP; Gene: near or holder gene ID; Effect: type of gene effects;–*log*_*10*_*P*_*EW*_: minus log experimental-wise P-value; %: estimated heritability for the effects; Gene Description: description of the candidate genes collected from NCBI gene database.
(PDF)

S4 Table Predicted genetic effects of individual and epistasis loci with standard error, significance, and heritability for BSA| Exer cofactor model. QTS: identified quantitative trait SNP; Gene: near or holder gene ID; Effect: type of gene effects;–*log*_*10*_*P*_*EW*_: minus log experimental-wise P-value; %: estimated heritability for the effects; Gene Description: description of the candidate genes collected from NCBI gene database.
(PDF)

S5 Table Predicted genetic effects of individual and epistasis loci with standard error, significance, and heritability for BSA| Smoke cofactor model. QTS: identified quantitative trait SNP; Gene: near or holder gene ID; Effect: type of gene effects;–*log*_*10*_*P*_*EW*_: minus log experimental-wise P-value; %: estimated heritability for the effects; Gene Description: description of the candidate genes collected from NCBI gene database.
(PDF)

S6 Table Predicted genetic effects of individual and epistasis loci with standard error, significance, and heritability for BSA| Trans cofactor model. QTS: identified quantitative trait SNP; Gene: near or holder gene ID; Effect: type of gene effects;–*log*_*10*_*P*_*EW*_: minus log experimental-wise P-value; %: estimated heritability for the effects; Gene Description: description of the candidate genes collected from NCBI gene database.
(PDF)

S7 Table Percentages of individuals carrying positive and negative effects corresponding to the identified loci using the base model. Base Model: QTS: identified quantitative trait SNP; Gene: near or holder gene ID; genetic effects for positive and negative of the identified loci including four ethnic groups: HA = Hispanic-American, AA = African-American, CA = Chinese-American, EA = European-American.
(PDF)

S8 Table Percentages of individuals carrying positive and negative effects corresponding to the additional loci identified using cofactor models. Cofactor Models: BSA|Exer = exercise cofactor model. BSA|Smoke = smoke cofactor model. BSA|Trans = transportation cofactor model. QTS: identified quantitative trait SNP; Gene: near or holder gene ID; genetic effects or positive and negative of the identified loci including four ethnic groups: HA = Hispanic-American, AA = African-American, CA = Chinese-American, EA = European-American.
(PDF)

S9 Table Ethnic-specific risk of cancer diseases. Information retrieved from: https://www.mdanderson.org/publications/cancerwise/race-and-ethnicity-as-cancer-risk-factors.h00-158592156.html.
(PDF)

S1 Text Statistical genetic models for unconditional and conditional genetic models. (PDF)
