# Supplementary material for: Genome-wide conditional association study reveals the influences of lifestyle cofactors on genetic regulation of body surface area in MESA population
Source: PLoS One. 2021 Jun 18;16(6):e0253167. doi: 10.1371/journal.pone.0253167 (PMC8213052; doi:10.1371/journal.pone.0253167)
Supplement: S1 Fig — EA = European- American, CA = Chinese-American, AA = African-American, and HA = Hispanic-American. (PDF) [file pone.0253167.s001.pdf]

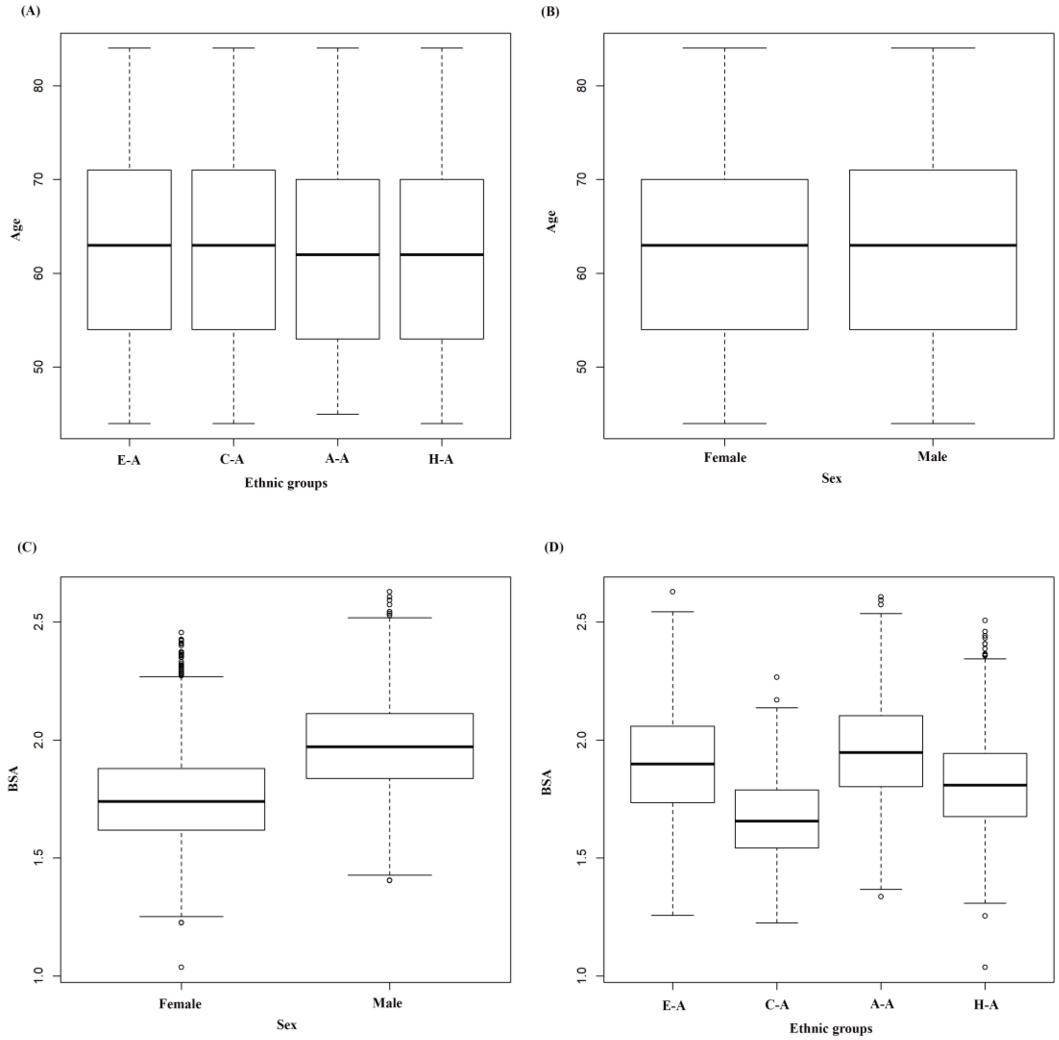

S1 Fig. Box plot for age, sex, and phenotypic distribution. EA = European- American, CA = Chinese-American, AA = African-American, and HA = Hispanic-American.
