# Supplementary material for: Genome-wide conditional association study reveals the influences of lifestyle cofactors on genetic regulation of body surface area in MESA population
Source: PLoS One. 2021 Jun 18;16(6):e0253167. doi: 10.1371/journal.pone.0253167 (PMC8213052; doi:10.1371/journal.pone.0253167)
Supplement: S2 Fig — Different colors used for indicating heritability due to different types of genetic effects. (PDF) [file pone.0253167.s002.pdf]

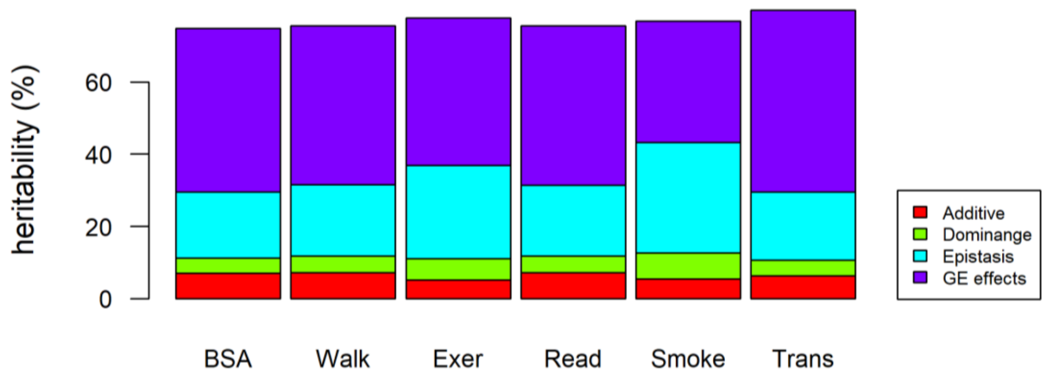

S2 Fig. Estimated heritability from base and cofactor models. Different colors used for indicating heritability due to different types of genetic effects.
