# Supplementary material for: Genome-wide conditional association study reveals the influences of lifestyle cofactors on genetic regulation of body surface area in MESA population
Source: PLoS One. 2021 Jun 18;16(6):e0253167. doi: 10.1371/journal.pone.0253167 (PMC8213052; doi:10.1371/journal.pone.0253167)
Supplement: S3 Fig — The size of the vertical axis is the genetic effects, and the horizontal axis is the SNP name and effects type. (PDF) [file pone.0253167.s003.pdf]

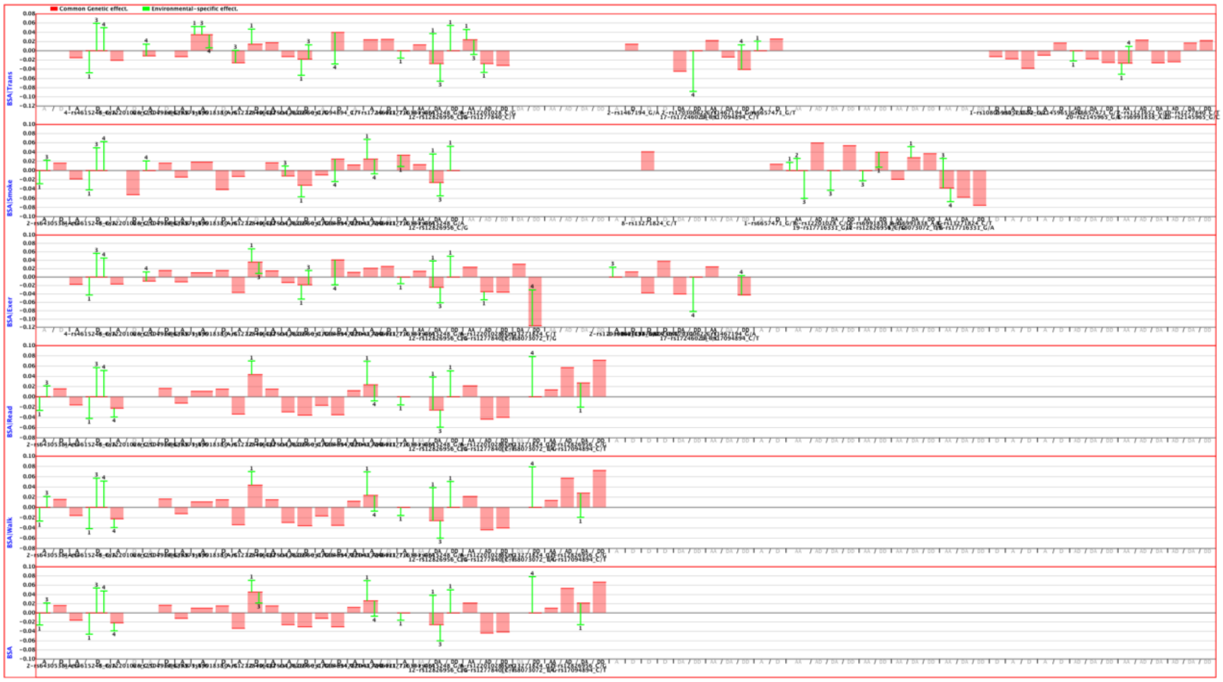

S3 Fig. Genetic and Genetic by Ethnic interaction effects plot for BSA base model and life-style cofactor models. The size of the vertical axis is the genetic effects, and the horizontal axis is the SNP name and effects type.
