# Supplementary material for: Genome-wide conditional association study reveals the influences of lifestyle cofactors on genetic regulation of body surface area in MESA population
Source: PLoS One. 2021 Jun 18;16(6):e0253167. doi: 10.1371/journal.pone.0253167 (PMC8213052; doi:10.1371/journal.pone.0253167)
Supplement: S4 Fig — (A) Genetic main effects of loci and (B) G × E effects of loci. The vertical axis for the size of genetic effects including four ethnic groups: HA = Hispanic-American, AA = African-American, CA = Chinese-American, EA = European-American; horizontal axis for the individual and epistasis loci; different color present different genetic effects according to a color scale, where gray color = no significant effects. (PDF) [file pone.0253167.s004.pdf]

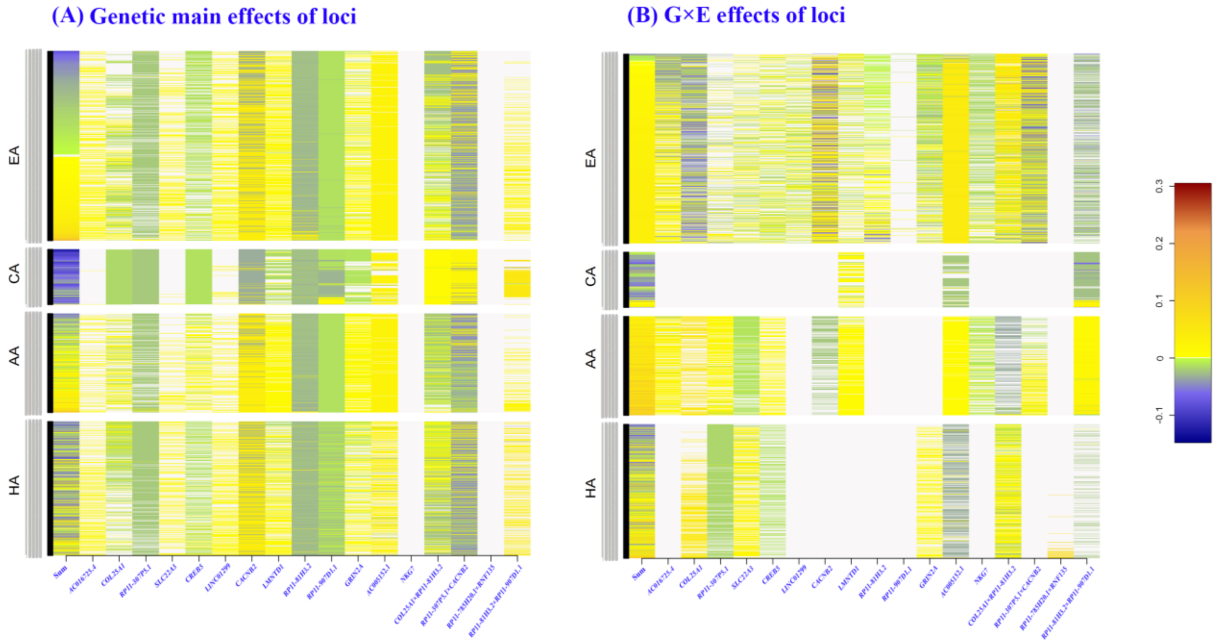

S4 Fig. Genetic effects matrix (G and GE) image plot of BSA loci. (A) Genetic main effects of loci and (B) G × E effects of loci. Vertical axis for the size of genetic effects including four ethnic groups: HA= Hispanic-American, AA= African-American, CA= Chinese-American, EA= European-American; horizontal axis for the individual and epistasis loci; different color present different genetic effects according to a color scale, where gray color = no significant effects.
