# Supplementary material for: Genome-wide conditional association study reveals the influences of lifestyle cofactors on genetic regulation of body surface area in MESA population
Source: PLoS One. 2021 Jun 18;16(6):e0253167. doi: 10.1371/journal.pone.0253167 (PMC8213052; doi:10.1371/journal.pone.0253167)
Supplement: S1 Table — CHR refers chromosome; SNP refers SNP ID; MAF refers minor allele frequency. (PDF) [file pone.0253167.s005.pdf]

S1 Table. Allele frequencies of the identified loci

CHR	SNP	Major allele	Minor allele	MAF
1	rs10801580	T	C	0.3059
1	rs6657471	G	T	0.05432
2	rs1467194	G	A	0.4128
2	rs1521652	G	C	0.2234
2	rs17030062	C	T	0.09683
2	rs6430538	A	G	0.301
4	rs4615248	G	A	0.4139
6	rs12201028	C	G	0.08815
6	rs2504934	G	A	0.1732
7	rs9639575	T	G	0.3279
8	rs13271824	C	T	0.1435
8	rs6991838	A	G	0.4522
10	rs1277840	C	T	0.4569
12	rs12826956	C	G	0.1668
12	rs6487504	A	G	0.2344
14	rs17094894	C	T	0.08828
16	rs4782041	A	G	0.3382
17	rs17246021	T	C	0.09289
17	rs8073072	T	G	0.2145
19	rs17716331	G	A	0.3528
20	rs2145965	G	C	0.4682

CHR refers chromosome; SNP refers SNP ID; MAF refers minor allele frequency.
