# Supplementary material for: Genome-wide conditional association study reveals the influences of lifestyle cofactors on genetic regulation of body surface area in MESA population
Source: PLoS One. 2021 Jun 18;16(6):e0253167. doi: 10.1371/journal.pone.0253167 (PMC8213052; doi:10.1371/journal.pone.0253167)
Supplement: S2 Table — QTS: identified quantitative trait SNP; Gene: near or holder gene ID; Effect: type of gene effects;–log10PEW: minus log experimental-wise P-value; %: estimated heritability for the effects; Gene Description: description of the candidate genes collected from NCBI gene database. (PDF) [file pone.0253167.s006.pdf]

S2 Table. Predicted genetic effects of individual and epistasis loci with standard error, significance, and heritability for BSA|Walk cofactor model

Chr_SNP_Allele	Gene	Effect	Estimate	SE	$-Log_{10}P_{EW}$	h^2(%)
2_rs6430538_A/G	*AC016725.4*	d	0.015	0.003	6.304	0.19
		ae_1	−0.027	0.004	13.298	0.98
		ae_3	0.021	0.004	6.909	
4_rs4615248_G/A	*COL25A1*	a	−0.016	0.002	12.409	0.45
		de_1	−0.042	0.004	27.567	2.12
		de_3	0.057	0.005	27.078	
		de_4	0.051	0.005	20.361	
6_rs12201028_C/G	*RP11–307P5.1*	a	−0.022	0.002	31.68	0.83
		ae_4	−0.017	0.004	5.187	0.49
6_rs2504934_G/A	*SLC22A3*	d	0.016	0.003	5.96	0.22
7_rs9639575_T/G	*CREB5*	a	−0.013	0.002	8.229	0.26
8_rs6991838_A/G	*CTD–3025N20.2*	a	0.010	0.002	5.423	0.17
		d	0.014	0.003	6.422	0.17
10_rs1277840_C/T	*CACNB2*	a	−0.034	0.002	47.373	1.89
		d	0.043	0.003	60.015	1.54
		de_1	0.026	0.004	11.055	0.49
12_rs6487504_A/G	*5.8kb 5' of IFLTD1*	a	0.015	0.002	12.591	0.37
12_rs12826956_C/G	*39kb 5' of RP11–81H3.2*	a	−0.029	0.002	47.073	1.44
		d	−0.036	0.003	26.277	1.08
14_rs17094894_C/T	*54kb 3' of RP11–907D1.1*	a	−0.017	0.002	18.603	0.46
		d	−0.036	0.005	12.447	1.06
16_rs4782041_A/G	*GRIN2A*	a	0.012	0.002	7.224	0.23
17_rs17246021_T/C	*AC005152.1*	a	0.024	0.002	35.486	0.92
		ae_1	0.045	0.003	60.221	1.53
		ae_4	−0.031	0.004	11.916	
19_rs17716331_G/A	*3.3kb 5' of NKG7*	ae_1	−0.016	0.003	5.692	0.32
4_rs4615248_G/A× 12_rs12826956_C/G	*COL25A1×* *39kb 5' of RP11–81H3.2*	da	−0.026	0.003	15.474	1.12
		dae_1	0.064	0.005	40.612	3.12
		dae_3	−0.034	0.006	8.071	
		dde_1	0.050	0.006	14.441	1.05
6_rs12201028_C/G× 10_rs1277840_C/T	*RP11–307P5.1×* *CACNB2*	aa	0.021	0.003	16.103	1.45
		ad	−0.044	0.003	51.572	3.19
		dd	−0.041	0.006	9.967	1.37
8_rs13271824_C/T× 17_rs8073072_T/G	*13kb 3' of RP11–785H20.1×* *24kb 3' of RNF135*	dde_4	0.079	0.015	6.694	5.16
12_rs12826956_C/G× 14_rs17094894_C/T	*39kb 5' of RP11–81H3.2×* *54kb 3' of RP11–907D1.1*	aa	0.013	0.002	8.604	0.56
		ad	0.057	0.005	25.848	5.37

		da	0.027	0.004	14.387	1.24
		dd	0.072	0.013	7.963	4.30
		dae_1	−0.047	0.005	24.021	2.50

QTS: identified quantitative trait SNP; Gene: near or holder gene ID; Effect: type of gene effects; $-log_{10}P_{EW}$: minus log experimental-wise *P*-value; h^2%: estimated heritability for the effects; Gene Description: description of the candidate genes collected from NCBI gene database.
