# Supplementary material for: Genome-wide conditional association study reveals the influences of lifestyle cofactors on genetic regulation of body surface area in MESA population
Source: PLoS One. 2021 Jun 18;16(6):e0253167. doi: 10.1371/journal.pone.0253167 (PMC8213052; doi:10.1371/journal.pone.0253167)
Supplement: S4 Table — QTS: identified quantitative trait SNP; Gene: near or holder gene ID; Effect: type of gene effects;–log10PEW: minus log experimental-wise P-value; %: estimated heritability for the effects; Gene Description: description of the candidate genes collected from NCBI gene database. (PDF) [file pone.0253167.s008.pdf]

S4 Table. Predicted genetic effects of individual and epistasis loci with standard error, significance, and heritability for BSA|Exer cofactor model

Chr_SNP_Allele	Gene	Effect	Estimate	SE	$-Log_{10}P_{EW}$	h^2(%)
2_rs17030062_C/T	*ACTR2*	ae_3	0.024	0.004	10.423	0.46
2_rs1467194_G/A	*TMEM163*	d	0.012	0.003	5.126	0.11
4_rs4615248_G/A	*COL25A1*	a	−0.018	0.002	14.179	0.47
		de_1	−0.043	0.004	29.241	1.79
		de_3	0.056	0.005	26.518	
		de_4	0.045	0.005	16.024	
6_rs12201028_C/G	*RP11–307P5.1*	a	−0.017	0.002	18.266	0.43
6_rs2504934_G/A	*SLC22A3*	a	−0.010	0.002	5.817	0.14
		d	0.016	0.003	5.593	0.19
		ae_4	0.022	0.004	6.117	0.39
7_rs9639575_T/G	*CREB5*	a	−0.012	0.002	7.827	0.23
8_rs6991838_A/G	*CTD–3025N20.2*	a	0.010	0.002	5.351	0.15
		d	0.015	0.003	7.073	0.17
8_rs13271824_C/T	*13kb 3' of RP11–785H20.1*	d	−0.038	0.004	22.866	1.12
10_rs1277840_C/T	*CACNB2*	a	−0.037	0.002	57.722	2.11
		d	0.035	0.003	40.344	0.93
		de_1	0.032	0.004	16.173	0.67
		de_3	−0.027	0.005	6.763	
12_rs6487504_A/G	*5.8kb 5' of IFLTD1*	a	0.015	0.002	12.223	0.33
12_rs12826956_C/G	*39kb 5' of RP11–81H3.2*	a	−0.014	0.002	11.162	0.29
		d	−0.019	0.003	8.081	0.28
		de_1	−0.034	0.005	13.152	0.79
		de_3	0.034	0.007	6.042	
14_rs17094894_C/T	*54kb 3' of RP11–907D1.1*	d	0.041	0.005	15.886	1.25
		de_4	−0.060	0.007	15.646	5.19
16_rs4782041_A/G	*GRIN2A*	a	0.011	0.002	6.186	0.17
17_rs8073072_T/G	*24kb 3' of RNF135*	d	0.038	0.005	15.827	1.07
17_rs17246021_T/C	*AC005152.1*	a	0.021	0.002	27.547	0.64
		d	0.025	0.005	7.19	0.47
19_rs17716331_G/A	*3.3kb 5' of NKG7*	ae_1	−0.016	0.003	5.828	0.29
2_rs17030062_C/T×<br>17_rs17246021_T/C	*ACTR2*×<br>*AC005152.1*	da	−0.041	0.006	12.208	2.52
		dde_4	−0.083	0.010	16.014	5.22
2_rs1467194_G/A×<br>14_rs17094894_C/T	*TMEM163*×<br>*54kb 3' of RP11–907D1.1*	aa	0.024	0.002	22.795	1.76
		dd	−0.044	0.008	8.235	1.44
		dde_4	0.047	0.010	5.738	1.30
4_rs4615248_G/A×<br>12_rs12826956_C/G	*COL25A1*×<br>*39kb 5' of RP11–81H3.2*	aa	0.014	0.003	6.629	0.55
		da	−0.025	0.003	14.759	0.97

		dae_1	0.063	0.005	40.028	2.98
		dae_3	−0.036	0.006	9.075	
		dde_1	0.050	0.006	14.165	0.95
6_rs12201028_C/G×<br>10_rs1277840_C/T	*RP11–307P5.1×<br>CACNB2*	*aa*	0.023	0.003	20.121	1.66
		ad	−0.036	0.003	34.692	1.93
		dd	−0.036	0.006	7.959	0.97
		ade_1	−0.019	0.004	5.017	0.54
8_rs13271824_C/T×<br>17_rs8073072_T/G	*13kb 3' of RP11–<br>785H20.1×<br>24kb 3' of RNF135*	*da*	0.030	0.004	13.207	1.38
		dd	−0.116	0.013	19.631	10.30
		dde_4	0.085	0.015	7.734	5.55

QTS: identified quantitative trait SNP; Gene: near or holder gene ID; Effect: type of gene effects; $-log_{10}P_{EW}$: minus log experimental-wise *P*-value; h^2%: estimated heritability for the effects; Gene Description: description of the candidate genes collected from NCBI gene database.
