# Supplementary material for: Genome-wide conditional association study reveals the influences of lifestyle cofactors on genetic regulation of body surface area in MESA population
Source: PLoS One. 2021 Jun 18;16(6):e0253167. doi: 10.1371/journal.pone.0253167 (PMC8213052; doi:10.1371/journal.pone.0253167)
Supplement: S5 Table — QTS: identified quantitative trait SNP; Gene: near or holder gene ID; Effect: type of gene effects;–log10PEW: minus log experimental-wise P-value; %: estimated heritability for the effects; Gene Description: description of the candidate genes collected from NCBI gene database. (PDF) [file pone.0253167.s009.pdf]

S5 Table. Predicted genetic effects of individual and epistasis loci with standard error, significance, and heritability for BSA|Smoke cofactor model

Chr_SNP_Allele	Gene	Effect	Estimate	SE	$-Log_{10}P_{EW}$	h^2(%)
1_rs6657471_G/T	*9.1kb 3' of RP4–771M4.3*	*d*	0.013	0.003	5.454	0.14
2_rs6430538_A/G	*AC016725.4*	*d*	0.016	0.003	6.586	0.19
		ae_1	−0.029	0.004	14.919	1.02
		ae_3	0.022	0.004	7.165	
4_rs4615248_G/A	*COL25A1*	*a*	−0.018	0.002	15.464	0.54
		de_1	−0.043	0.004	28.53	2.14
		de_3	0.049	0.005	20.667	
		de_4	0.062	0.005	29.345	
6_rs12201028_C/G	*RP11–307P5.1*	*d*	−0.053	0.004	33.256	2.19
6_rs2504934_G/A	*SLC22A3*	*d*	0.016	0.003	5.558	0.19
		ae_4	0.020	0.004	5.497	0.34
7_rs9639575_T/G	*CREB5*	*a*	−0.014	0.002	10.495	0.32
8_rs6991838_A/G	*CTD–3025N20.2*	*a*	0.018	0.002	15.099	0.49
		d	−0.041	0.003	47.687	1.35
8_rs13271824_C/T	*13kb 3' of RP11–785H20.1*	*d*	0.040	0.004	24.973	1.27
10_rs1277840_C/T	*CACNB2*	*a*	−0.013	0.002	7.883	0.27
12_rs6487504_A/G	*5.8kb 5' of IFLTD1*	*a*	0.016	0.002	14.847	0.42
12_rs12826956_C/G	*39kb 5' of RP11–81H3.2*	*a*	−0.012	0.002	8.715	0.23
		d	−0.032	0.003	21.001	0.81
		ae_3	0.021	0.004	6.587	0.44
		de_1	−0.026	0.005	7.922	0.49
14_rs17094894_C/T	*54kb 3' of RP11–907D1.1*	*a*	−0.010	0.002	7.227	0.16
		d	0.024	0.005	6.071	0.46
		de_4	−0.048	0.007	10.541	4.24
16_rs4782041_A/G	*GRIN2A*	*a*	0.011	0.002	6.778	0.20
17_rs17246021_T/C	*AC005152.1*	*a*	0.025	0.002	38.995	0.96
		ae_1	0.042	0.003	52.947	1.40
		ae_4	−0.032	0.004	12.701	
19_rs17716331_G/A	*3.3kb 5' of NKG7*	*a*	0.033	0.002	52.784	1.72
		ae_1	−0.025	0.003	12.028	0.45
4_rs4615248_G/A×<br>12_rs12826956_C/G	*COL25A1×*<br>*39kb 5' of RP11–81H3.2*	*aa*	0.013	0.003	5.804	0.50
		da	−0.027	0.003	16.317	1.13
		dae_1	0.062	0.005	38.927	3.72
		dae_3	−0.029	0.006	5.898	
		dde_1	0.052	0.006	15.545	1.58
6_rs12201028_C/G×<br>19_rs17716331_G/A	*RP11–307P5.1×*<br>*3.3kb 5' of NKG7*	*ad*	0.059	0.003	72.818	5.55
		dd	0.054	0.007	15.121	2.28

		aae_1	0.017	0.004	5.24	3.78
		aae_2	0.026	0.005	6.708	
		aae_3	−0.060	0.005	29.618	
		dae_3	−0.043	0.009	5.382	2.45
8_rs6991838_A/G× 12_rs12826956_C/G	*CTD–3025N20.2× 39kb 5' of RP11–81H3.2*	*dd*	0.039	0.005	13.171	1.22
		aae_3	−0.023	0.005	6.077	0.85
		dde_1	−0.032	0.007	6.115	1.87
8_rs6991838_A/G× 17_rs8073072_T/G	*CTD–3025N20.2× 24kb 3' of RNF135*	*aa*	−0.019	0.002	15.599	1.19
		da	0.028	0.003	19.05	1.21
		dd	0.037	0.008	5.592	1.06
		dae_1	0.023	0.004	8.611	1.05
8_rs13271824_C/T× 19_rs17716331_G/A	*13kb 3' of RP11–785H20.1×* 19_rs17716331_G/A	*aa*	−0.038	0.002	54.928	4.68
		da	−0.058	0.004	38.417	5.35
		dd	−0.076	0.008	22.913	4.53
		aae_3	0.064	0.005	40.255	4.33
		aae_4	−0.030	0.006	7.141	

QTS: identified quantitative trait SNP; Gene: near or holder gene ID; Effect: type of gene effects; $-log_{10}P_{EW}$: minus log experimental-wise P-value; h^2%: estimated heritability for the effects; Gene Description: description of the candidate genes collected from NCBI gene database.
