# Supplementary material for: Genome-wide conditional association study reveals the influences of lifestyle cofactors on genetic regulation of body surface area in MESA population
Source: PLoS One. 2021 Jun 18;16(6):e0253167. doi: 10.1371/journal.pone.0253167 (PMC8213052; doi:10.1371/journal.pone.0253167)
Supplement: S6 Table — QTS: identified quantitative trait SNP; Gene: near or holder gene ID; Effect: type of gene effects;–log10PEW: minus log experimental-wise P-value; %: estimated heritability for the effects; Gene Description: description of the candidate genes collected from NCBI gene database. (PDF) [file pone.0253167.s010.pdf]

S6 Table. Predicted genetic effects of individual and epistasis loci with standard error, significance, and heritability for BSA|Trans cofactor model

Chr_SNP_Allele	Gene	Effect	Estimate	SE	$-Log_{10}P_{EW}$	h^2 (%)
1_rs6657471_G/T	*9.1kb 3' of RP4–771M4.3*	*d*	0.025	0.003	17.585	0.44
		ae_1	0.020	0.003	8.547	0.37
1_rs10801580_T/C	*CFHR2*	*d*	−0.013	0.003	5.142	0.12
2_rs1467194_G/A	*TMEM163*	*d*	0.014	0.003	7.102	0.14
2_rs1521652_G/C	*ERBB4*	*a*	−0.018	0.002	19.737	0.47
		d	−0.038	0.004	27.734	1.04
4_rs4615248_G/A	*COL25A1*	*a*	−0.016	0.002	11.738	0.35
		de_1	−0.048	0.004	36.492	1.93
		de_3	0.059	0.005	29.756	
		de_4	0.049	0.005	19.388	
6_rs12201028_C/G	*RP11–307P5.1*	*a*	−0.021	0.002	29.559	0.64
6_rs2504934_G/A	*SLC22A3*	*a*	−0.012	0.002	8.623	0.20
		ae_4	0.026	0.004	8.577	0.51
7_rs9639575_T/G	*CREB5*	*a*	−0.013	0.002	8.649	0.23
8_rs6991838_A/G	*CTD–3025N20.2*	*a*	0.035	0.002	56.609	1.68
		ae_1	0.018	0.004	5.686	0.67
		ae_3	0.018	0.004	5.001	
		ae_4	−0.029	0.005	8.564	
10_rs1277840_C/T	*CACNB2*	*a*	−0.026	0.002	29.926	0.97
		d	0.014	0.003	6.781	0.13
		ae_3	0.027	0.005	7.65	0.64
		de_1	0.033	0.004	17.378	0.55
12_rs6487504_A/G	*5.8kb 5' of IFLTD1*	*a*	0.017	0.002	16.714	0.41
12_rs12826956_C/G	*39kb 5' of RP11–81H3.2*	*a*	−0.013	0.002	10.556	0.25
		d	−0.019	0.003	8.04	0.25
		de_1	−0.034	0.005	13.718	0.67
		de_3	0.031	0.007	5.31	
14_rs17094894_C/T	*54kb 3' of RP11–907D1.1*	*d*	0.039	0.005	15.287	1.08
		de_4	−0.068	0.007	20.612	6.74
17_rs17246021_T/C	*AC005152.1*	*a*	0.023	0.002	35.163	0.75
		d	0.024	0.005	7.049	0.41
19_rs17716331_G/A	*3.3kb 5' of NKG7*	ae_1	−0.016	0.003	5.825	0.24
20_rs2145965_G/C	*29kb 5' of RP5–1177M21.1*	*a*	−0.010	0.002	5.012	0.14
		d	0.016	0.003	8.978	0.18
1_rs6657471_G/T× 20_rs2145965_G/C	*9.1kb 3' of RP4–771M4.3× 29kb 5' of RP5–1177M21.1*	*da*	−0.018	0.004	5.549	0.47
		dd	−0.026	0.004	8.71	0.46
		ade_1	−0.023	0.005	5.749	0.56

2_rs17030062_C/T×<br>17_rs17246021_T/C	*ACTR2*×<br>*AC005152.1*	*da*	−0.045	0.006	14.73	2.79
		dde_4	−0.088	0.010	18.375	8.30
2_rs1467194_G/A×<br>14_rs17094894_C/T	*TMEM163*×<br>*54kb 3' of RP11–907D1.1*	*aa*	0.022	0.002	19.297	1.34
		da	−0.014	0.003	6.007	0.27
		dd	−0.041	0.007	7.42	1.16
		dde_4	0.053	0.010	7.259	1.43
2_rs1521652_G/C×<br>8_rs6991838_A/G	*ERBB4*×<br>*CTD–3025N20.2*	*aa*	−0.027	0.003	27.342	2.10
		ad	0.023	0.003	12.094	0.74
		da	−0.027	0.004	9.083	1.00
		aae_1	−0.024	0.004	8.428	2.70
		aae_4	0.037	0.006	9.09	
4_rs4615248_G/A×<br>12_rs12826956_C/G	*COL25A1*×<br>*39kb 5' of RP11–81H3.2*	*aa*	0.012	0.003	5.655	0.42
		da	−0.028	0.003	18.742	1.13
		dae_1	0.065	0.005	43.534	2.81
		dae_3	−0.038	0.006	9.97	
		dde_1	0.054	0.006	16.99	1.03
6_rs12201028_C/G×<br>10_rs1277840_C/T	*RP11–307P5.1*×<br>*CACNB2*	*aa*	0.023	0.003	20.124	1.50
		ad	−0.028	0.003	22.473	1.11
		dd	−0.032	0.006	6.769	0.74
		aae_1	0.023	0.004	7.589	1.57
		aae_3	−0.032	0.006	8.044	
		ade_1	−0.019	0.004	5.253	0.32
10_rs1277840_C/T×<br>20_rs2145965_G/C	*CACNB2*×<br>*29kb 5' of RP5–1177M21.1*	*ad*	−0.024	0.004	10.504	0.81
		da	0.017	0.004	5.545	0.38
		dd	0.022	0.004	8.043	0.33

QTS: identified quantitative trait SNP; Gene: near or holder gene ID; Effect: type of gene effects; $-log_{10}P_{EW}$: minus log experimental-wise P-value; h^2%: estimated heritability for the effects; Gene Description: description of the candidate genes collected from NCBI gene database.
