# Supplementary material for: Genome-wide conditional association study reveals the influences of lifestyle cofactors on genetic regulation of body surface area in MESA population
Source: PLoS One. 2021 Jun 18;16(6):e0253167. doi: 10.1371/journal.pone.0253167 (PMC8213052; doi:10.1371/journal.pone.0253167)
Supplement: S7 Table — Base Model: QTS: identified quantitative trait SNP; Gene: near or holder gene ID; genetic effects for positive and negative of the identified loci including four ethnic groups: HA = Hispanic-American, AA = African-American, CA = Chinese-American, EA = European-American. (PDF) [file pone.0253167.s011.pdf]

**S7 Table. Percentages of individuals carrying positive and negative effects corresponding to the identified loci using base model**

| Chr_SNP_Allele | Gene | EA | | CA | | AA | | HA | |
|---|---|---|---|---|---|---|---|---|---|
| | | Positive | Negative | Positive | Negative | Positive | Negative | Positive | Negative |
| 2_rs6430538_A/G | *AC016725.4* | 74.41 | 25.59 | 1.04 | 0.00 | 97.92 | 2.08 | 39.36 | 0.00 |
| 4_rs4615248_G/A | *COL25A1* | 20.77 | 79.23 | 0.00 | 99.41 | 82.69 | 17.31 | 59.82 | 40.18 |
| 6_rs12201028_C/G | *RP11–307P5.1* | 0.90 | 80.46 | 0.00 | 99.55 | 1.76 | 75.24 | 0.25 | 87.56 |
| 6_rs2504934_G/A | *SLC22A3* | 34.91 | 0.00 | 0.74 | 0.00 | 20.76 | 78.93 | 92.27 | 5.73 |
| 7_rs9639575_T/G | *CREB5* | 5.39 | 56.34 | 0.00 | 99.26 | 88.46 | 11.54 | 9.74 | 90.26 |
| 8_rs6991838_A/G | *LINC01299* | 48.68 | 0.00 | 11.28 | 0.00 | 23.96 | 0.00 | 45.66 | 0.00 |
| 10_rs1277840_C/T | *CACNB2* | 69.96 | 30.04 | 8.46 | 91.54 | 87.26 | 12.74 | 75.61 | 24.39 |
| 12_rs6487504_A/G | *5.8kb 5' of LMNTD1* | 72.19 | 1.51 | 9.35 | 49.26 | 82.69 | 0.64 | 53.44 | 9.66 |
| 12_rs12826956_C/G | *39kb 5' of RP11–81H3.2* | 5.58 | 94.42 | 0.45 | 99.55 | 2.24 | 97.76 | 2.13 | 97.87 |
| 14_rs17094894_C/T | *54kb 3' of RP11–907D1.1* | 0.00 | 100.00 | 13.35 | 86.65 | 0.00 | 100.00 | 3.44 | 96.56 |
| 16_rs4782041_A/G | *GRIN2A* | 47.82 | 52.18 | 3.26 | 65.73 | 82.45 | 1.28 | 83.80 | 16.20 |
| 17_rs17246021_T/C | *AC005152.1* | 96.36 | 0.00 | 63.80 | 4.75 | 95.03 | 0.16 | 6.30 | 60.23 |
| 19_rs17716331_G/A | *3.3kb 5' of NKG7* | 9.32 | 51.47 | 0.00 | 0.00 | 9.62 | 49.52 | 0.00 | 0.00 |
| 4_rs4615248_G/A× 12_rs12826956_C/G | *COL25A1× 39kb 5' of RP11–81H3.2* | 76.68 | 23.32 | 98.96 | 1.04 | 18.91 | 81.09 | 40.34 | 59.66 |
| 6_rs12201028_C/G× 10_rs1277840_C/T | *RP11–307P5.1× CACNB2* | 30.27 | 69.73 | 91.54 | 8.46 | 14.02 | 85.98 | 24.47 | 75.53 |
| 8_rs13271824_C/T× 17_rs8073072_T/G | *13kb 3' of RP11–785H20.1× 24kb 3' of RNF135* | 0.00 | 0.00 | 0.00 | 0.00 | 0.00 | 0.00 | 5.32 | 0.00 |
| 12_rs12826956_C/G× 14_rs17094894_C/T | *39kb 5' of RP11–81H3.2× 54kb 3' of RP11–907D1.1* | 0.28 | 35.62 | 64.84 | 35.16 | 97.76 | 2.24 | 41.98 | 1.06 |

**Base Model:** QTS: identified quantitative trait SNP; Gene: near or holder gene ID; genetic effects for positive and negative of the identified loci including four ethnic groups: HA= Hispanic-American, AA= African-American, CA= Chinese-American, EA= European-American.
