# Supplementary material for: Genome-wide conditional association study reveals the influences of lifestyle cofactors on genetic regulation of body surface area in MESA population
Source: PLoS One. 2021 Jun 18;16(6):e0253167. doi: 10.1371/journal.pone.0253167 (PMC8213052; doi:10.1371/journal.pone.0253167)
Supplement: S8 Table — Cofactor Models: BSA|Exer = exercise cofactor model. BSA|Smoke = smoke cofactor model. BSA|Trans = transportation cofactor model. QTS: identified quantitative trait SNP; Gene: near or holder gene ID; genetic effects or positive and negative of the identified loci including four ethnic groups: HA = Hispanic-American, AA = African-American, CA = Chinese-American, EA = European-American. (PDF) [file pone.0253167.s012.pdf]

S8 Table. Percentages of individuals carrying positive and negative effects corresponding to the additional loci identified using cofactor models

Cofactor	Chr_SNP_Allele	Gene	EA		CA		AA		HA	
			Positive	Negative	Positive	Negative	Positive	Negative	Positive	Negative
BSA|Exer	2_rs17030062_C/T	ACTR2	2.37	0.00	50.30	0.00	100.00	0.00	34.29	65.71
	2_rs1467194_G/A	TMEM163	44.42	0.00	35.31	0.00	32.45	0.00	48.85	0.00
	8_rs13271824_C/T	13kb 3' of RP11–785H20.1	0.00	12.82	0.00	52.08	0.00	5.13	0.00	32.32
	2_rs17030062_C/T× 17_rs17246021_T/C	ACTR2× AC005152.1	0.00	5.72	6.68	43.32	0.00	6.25	20.29	29.38
	2_rs1467194_G/A× 14_rs17094894_C/T	TMEM163× 54kb 3' of RP11–907D1.1	36.71	63.29	17.95	82.05	61.70	38.06	40.02	48.28
	8_rs13271824_C/T× 17_rs8073072_T/G	13kb 3' of RP11–785H20.1× 24kb 3' of RNF135	12.54	2.98	51.78	0.30	0.48	40.14	27.00	18.66
BSA|Smoke	1_rs6657471_G/T	9.1kb 3' of RP4–771M4.3	40.44	0.00	2.37	0.00	41.91	0.00	37.97	0.00
	8_rs13271824_C/T	13kb 3' of RP11–785H20.1	12.82	0.00	52.08	0.00	5.13	0.00	32.32	0.00
	8_rs6991838_A/G× 12_12826956_C/G	CTD-3025N20.2× 39kb5' of RP11–81H3.2	57.62	9.22	11.13	8.16	46.31	53.69	70.13	29.87
	8_rs6991838_A/G× 17_rs8073072_T/G	CTD–3025N20.2× 24kb 3' of RNF135	67.98	32.02	99.85	0.15	66.19	33.81	74.96	25.04
	8_rs13271824_C/T× 19_rs17716331_G/A	13kb 3' of RP11–785H20.1× 3.3kb 5' of NKG7	9.65	55.68	21.51	76.71	11.62	49.20	12.85	61.46
BSA|Trans	1_rs6657471_G/T	9.1kb 3' of RP4–771M4.3	93.38	6.6	2.37	0.00	90.63	9.38	37.97	0.00
	1_rs10801580_T/C	CFHR2	0.00	36.66	0.00	36.20	0.00	14.10	0.00	46.48
	2_rs1467194_G/A	TMEM163	44.42	0.00	35.31	0.00	32.45	0.00	48.85	0.00
	2_rs1521652_G/C	ERBB4	0.85	99.15	61.13	38.87	0.72	99.28	7.94	92.06
	20_rs2145965_G/C	29kb 5' of RP5–1177M21.1	49.67	0.00	19.29	0.00	0.00	36.78	49.84	0.00
	1_rs6657471_G/T× 20_rs2145965_G/C	9.1kb 3' of RP4–771M4.3× 29kb 5' of RP5–1177M21.1	11.07	59.41	0.15	2.23	15.63	84.38	27.58	41.98
	2_rs17030062_C/T× 17_rs17246021_T/C	ACTR2× AC005152.1	0.14	5.72	22.55	43.32	93.59	6.41	25.45	74.55
	2_rs1467194_G/A× 14_rs17094894_C/T	TMEM163× 54kb 3' of RP11–907D1.1	36.71	63.29	17.95	82.05	61.70	38.06	40.02	48.28
	2_rs1521652_G/C× 8_rs6991838_A/G	ERBB4× CTD–3025N20.2	67.27	32.73	39.02	60.98	27.96	72.04	68.41	31.59
	10_rs1277840_C/T× 20_rs2145965_G/C	CACNB2× 29kb 5' of RP5–1177M21.1	50.14	23.75	8.16	17.80	57.93	42.07	51.80	23.24

Cofactor Models: BSA|Exer = exercise cofactor model. BSA|Smoke = smoke cofactor model. BSA|Trans = transportation cofactor model. QTS: identified quantitative trait SNP; Gene: near or holder gene ID; genetic effects

or positive and negative of the identified loci including four ethnic groups: HA= Hispanic-American, AA= African-American, CA= Chinese-American, EA= European-American.
