# Supplementary material for: Genome-wide conditional association study reveals the influences of lifestyle cofactors on genetic regulation of body surface area in MESA population
Source: PLoS One. 2021 Jun 18;16(6):e0253167. doi: 10.1371/journal.pone.0253167 (PMC8213052; doi:10.1371/journal.pone.0253167)
Supplement: S9 Table — Information retrieved from: https://www.mdanderson.org/publications/cancerwise/race-and-ethnicity-as-cancer-risk-factors.h00-158592156.html. (PDF) [file pone.0253167.s013.pdf]

S9 Table. Ethnic specific risk of cancer diseases

	Lung Cancer - Men and Women	Prostate Cancer – Men	Breast Cancer - Women
Highest Incidence Rate	African-American males	African- American	White, non-Hispanic
Lowest Incidence Rate	Hispanic females	American Indian/Natives	Korean American
Highest Death Rate	African-American males	African- American	African-American
Lowest Death Rate	Hispanic females	Asian / Pacific Islander	Chinese American

Information retrieved from: https://www.mdanderson.org/publications/cancerwise/race-and-ethnicity-as-cancer-risk-factors.h00-158592156.html
