# Supplementary material for: Genome-wide conditional association study reveals the influences of lifestyle cofactors on genetic regulation of body surface area in MESA population
Source: PLoS One. 2021 Jun 18;16(6):e0253167. doi: 10.1371/journal.pone.0253167 (PMC8213052; doi:10.1371/journal.pone.0253167)
Supplement: S1 Text — (PDF) [file pone.0253167.s014.pdf]

S1 Text: Statistical genetic models for unconditional and conditional genetic models

Unconditional genetic model for GWAS analysis is

$$y_{hk} = \mu + s_{hk} + \sum_i a_i x_{A_{ik}} + \sum_i d_i x_{D_{ik}} + \sum_{i<j} aa_{ij} x_{AA_{ik}} + \sum_{i<j} ad_{ij} x_{AD_{ijk}} + \sum_{i<j} da_{ij} x_{DA_{ijk}} + \sum_{i<j} dd_{ij} x_{DD_{ijk}}$$
$$+ e_h + \sum_i ae_{ih} u_{AE_{ihk}} + \sum_i de_{ih} u_{DE_{ihk}} + \sum_i aae_{ih} u_{AAE_{ihk}} + \sum_i ade_{ih} u_{ADE_{ihk}} + \sum_i dae_{ih} u_{DAE_{ihk}} + \sum_i dde_{ih} u_{DDE_{ihk}} + \varepsilon_{hk}$$

Conditional genetic model given walking status is

$$y_{hk} = \mu + s_{hk} + walk_{hk} + \sum_i a_i x_{A_{ik}} + \sum_i d_i x_{D_{ik}} + \sum_{i<j} aa_{ij} x_{AA_{ik}} + \sum_{i<j} ad_{ij} x_{AD_{ijk}} + \sum_{i<j} da_{ij} x_{DA_{ijk}} + \sum_{i<j} dd_{ij} x_{DD_{ijk}}$$
$$+ e_h + \sum_i ae_{ih} u_{AE_{ihk}} + \sum_i de_{ih} u_{DE_{ihk}} + \sum_i aae_{ih} u_{AAE_{ihk}} + \sum_i ade_{ih} u_{ADE_{ihk}} + \sum_i dae_{ih} u_{DAE_{ihk}} + \sum_i dde_{ih} u_{DDE_{ihk}} + \varepsilon_{hk}$$

Here, $walk_{hk}$ is moderate walking (walking to get places to the bus, car, work, into the store; minute/week) of the kth individual in the hth ethnic group.

Conditional genetic model given exercise status is

$$y_{hk} = \mu + s_{hk} + exercise_{hk} + \sum_i a_i x_{A_{ik}} + \sum_i d_i x_{D_{ik}} + \sum_{i<j} aa_{ij} x_{AA_{ik}} + \sum_{i<j} ad_{ij} x_{AD_{ijk}} + \sum_{i<j} da_{ij} x_{DA_{ijk}} + \sum_{i<j} dd_{ij} x_{DD_{ijk}}$$
$$+ e_h + \sum_i ae_{ih} u_{AE_{ihk}} + \sum_i de_{ih} u_{DE_{ihk}} + \sum_i aae_{ih} u_{AAE_{ihk}} + \sum_i ade_{ih} u_{ADE_{ihk}} + \sum_i dae_{ih} u_{DAE_{ihk}} + \sum_i dde_{ih} u_{DDE_{ihk}} + \varepsilon_{hk}$$

Here, $exercise_{hk}$ is moderate walking exercise (min/wk M ~ Su) of the kth individual in the hth ethnic group.

Conditional genetic model given reading status is

$$y_{hk} = \mu + s_{hk} + read_{hk} + \sum_i a_i x_{A_{ik}} + \sum_i d_i x_{D_{ik}} + \sum_{i<j} aa_{ij} x_{AA_{ik}} + \sum_{i<j} ad_{ij} x_{AD_{ijk}} + \sum_{i<j} da_{ij} x_{DA_{ijk}} + \sum_{i<j} dd_{ij} x_{DD_{ijk}}$$
$$+ e_h + \sum_i ae_{ih} u_{AE_{ihk}} + \sum_i de_{ih} u_{DE_{ihk}} + \sum_i aae_{ih} u_{AAE_{ihk}} + \sum_i ade_{ih} u_{ADE_{ihk}} + \sum_i dae_{ih} u_{DAE_{ihk}} + \sum_i dde_{ih} u_{DDE_{ihk}} + \varepsilon_{hk}$$

Here, $read_{hk}$ is Light Leisure Read (Read, knit, sew, visit, do nothing, non-work recreational computer; minute/week) of the kth individual in the hth ethnic group.

Conditional genetic model given smoking status is

$$y_{hk} = \mu + s_{hk} + smoke_{hk} + \sum_i a_i x_{A_{ik}} + \sum_i d_i x_{D_{ik}} + \sum_{i<j} aa_{ij} x_{AA_{ik}} + \sum_{i<j} ad_{ij} x_{AD_{ijk}} + \sum_{i<j} da_{ij} x_{DA_{ijk}} + \sum_{i<j} dd_{ij} x_{DD_{ijk}}$$
$$+ e_h + \sum_i ae_{ih} u_{AE_{ihk}} + \sum_i de_{ih} u_{DE_{ihk}} + \sum_i aae_{ih} u_{AAE_{ihk}} + \sum_i ade_{ih} u_{ADE_{ihk}} + \sum_i dae_{ih} u_{DAE_{ihk}} + \sum_i dde_{ih} u_{DDE_{ihk}} + \varepsilon_{hk}$$

Here, $smoke_{hk}$ is Pack-Years of cigarette smoking of the kth individual in the hth ethnic group.

Conditional genetic model given transportation status is

$$y_{hk} = \mu + s_{hk} + transportation_{hk} + \sum_i a_i x_{A_{ik}} + \sum_i d_i x_{D_{ik}} + \sum_{i<j} aa_{ij} x_{AA_{ik}} + \sum_{i<j} ad_{ij} x_{AD_{ijk}} + \sum_{i<j} da_{ij} x_{DA_{ijk}} + \sum_{i<j} dd_{ij} x_{DD_{ijk}}$$
$$+ e_h + \sum_i ae_{ih} u_{AE_{ihk}} + \sum_i de_{ih} u_{DE_{ihk}} + \sum_i aae_{ih} u_{AAE_{ihk}} + \sum_i ade_{ih} u_{ADE_{ihk}} + \sum_i dae_{ih} u_{DAE_{ihk}} + \sum_i dde_{ih} u_{DDE_{ihk}} + \varepsilon_{hk}$$

Here, $transportation_{hk}$ is light transportation (drive or ride in car, ride the bus/subway, including travel to work; minute/week) of the kth individual in the hth ethnic group.
